# miRNA transcriptome and myofiber characteristics of lamb skeletal muscle during hypertrophic growth^1^

**DOI:** 10.3389/fgene.2022.988756

**Published:** 2022-08-30

**Authors:** M. A. Greene, R. Powell, T. Bruce, W. C. Bridges, S. K. Duckett

**Affiliations:** ^1^ Department of Animal and Veterinary Sciences, Clemson University, Clemson, SC, United States; ^2^ Clemson Light Imaging Facility, Clemson University, Clemson, SC, United States; ^3^ Department of Bioengineering, Clemson University, Clemson, SC, United States; ^4^ School of Mathematical and Statistical Sciences, Clemson University, Clemson, SC, United States

**Keywords:** miRNA, skeletal muscle, transcriptome (RNA-seq), hypertrophy, sheep—lamb

## Abstract

Postnatal muscle growth is achieved through hypertrophy of the muscle fibers and is impacted by the activity of satellite cells, the quiescent muscle stem cell. Several miRNAs are preferentially expressed in skeletal muscle and could provide a mechanism for increasing muscle hypertrophy through satellite cell proliferation and/or differentiation. The objectives of this study were to: 1) Characterize the miRNA transcriptome of the longissimus thoracis et lumborum muscle at several developmental timepoints [gestational d 85 (PN1), 110 (PN2), 133 (PN3), postnatal d 42 (PW1), 65 (PW2), 243 (MAT)] during muscle hypertrophy in lambs, and 2) examine miR-29a, identified in sequencing to be differentially regulated across development, loss of function on satellite cell proliferation and differentiation. Muscle fiber characteristics showed drastic increases (*p* < 0.0001) in fiber size and alterations in muscle fiber type occur during pre and postnatal development. miRNA sequencing comparisons were performed in developmental order (PN1 vs. PN2, PN2 vs. PN3, PN3 vs. PW1, PW1 vs. PW2, PW2 vs. MAT). There were 184 differentially expressed (*P*_*adj*_ < 0.05) miRNA, 142 unique miRNA, from all 5 comparisons made. The transitional stage (PN3 vs. PW1) had the largest number (115) of differentially expressed miRNA. Inhibition of miR-29a in satellite cell culture increased (*p* < 0.05) cell proliferation and differentiation capacity. Characterization of the miRNA transcriptome provides valuable insights into the miRNA involved in muscle fiber hypertrophy and the potential importance of the transitional period.

## Introduction

Adult muscle tissue is composed of multinucleated myofibers, or muscle fibers, that originate from mesenchymal stems cells and the mesodermal layer of the embryo (Houba and Te Pas, 2004). Muscle fiber development for sheep consists of a prenatal hyperplasia stage, complete between gestational d (gd) 78 (Zhu et al., 2004) and gd85 (Fahey et al., 2005) in the sheep, at which point muscle growth occurs *via* hypertrophy. Postnatal muscle growth is achieved through hypertrophy of the muscle fibers and is impacted by the activity of satellite cells, the quiescent muscle stem cell (Mauro, 1961; Rehfeldt et al., 2011; Chal and Pourquié, 2017). Satellite cells, when activated, will proliferate and differentiate to fuse with existing myofibers to promote hypertrophy, replace nuclei that are no longer functional, or repair damage to the muscle fiber (Cardasis and Cooper, 1975). During neonatal growth, there is a rapid accumulation of protein and myonuclei in skeletal muscle related to greater satellite cell numbers during this time period that decline with age (Davis and Fiorotto, 2009). Several molecular pathways that regulate the hypertrophy process in skeletal muscle have been documented and include IGF1, myostatin, androgens, B-agonists and osteocalcin (Schiaffino et al., 2021). However, many factors appear involved in skeletal muscle hypertrophy but little is known about the role of non-coding RNAs in this process.

miRNA are small noncoding RNA that regulate 60% of protein coding gene expression post transcriptionally in the human genome (Friedman et al., 2009). miRNA usually repress gene expression through complementary binding with target mRNA 3’ untranslated regions (Hu and Coller, 2012). Hundreds of miRNA have been characterized across numerous cell lines, the majority of which focus on cancer and tumor formation/progression, or in other words cellular proliferation (Forterre et al., 2020). Several miRNAs are preferentially expressed in skeletal muscle and are involved in muscle development (Horak et al., 2016). miRNAs could provide a mechanism for increasing muscle hypertrophy through satellite cell proliferation and/or differentiation (Rupaimoole and Slack, 2017). Several *in vitro* studies have shown that certain miRNA can alter myocyte proliferation and differentiation through the targeting of mRNA (Anderson et al., 2006; Chen et al., 2006; Crist et al., 2012; Antoniou et al., 2014; Qadir et al., 2014). The use of miRNA mimics or inhibitors has been shown to alter satellite cell proliferation *in vitro* with miR-27b in sheep (Zhang et al., 2018), with miR-192 (Zhao et al., 2016) and miR-199b in pigs (Zhu et al., 2019), and with miR-92a in mice (Verma et al., 2019), which indicate that miRNA may play a role in skeletal muscle hypertrophy. The objectives of this study were to: 1) Characterize the miRNA transcriptome of the longissimus thoracis et lumborum muscle at several developmental timepoints (gd 85, 110, 133, postnatal d 42, 65, 243) during muscle hypertrophy in lambs, and 2) examine miR-29a, identified in sequencing to be differentially regulated across development, loss of function on satellite cell proliferation and differentiation.

## Materials and methods

All animal experimental procedures were reviewed and approved by the Clemson University Institutional Animal Care and Use Committee (AUP-2018-055 and AUP-2018-049).

### Experimental design

Suffolk ewes (*n* = 22) were mated to a single Texel ram (Texel Muscled; GeneSeek). Ewes were confirmed pregnant at gd 65 by transabdominal ultrasound (BCF Easi-Scan Curve; MIV Imaging, Rochester MN). At gd 85 (PN1), 110 (PN2), and 133 (PN3) of gestation, terminal surgeries (n = 3/time) were performed, and fetuses collected. Fetuses were towel dried and weighed and the right-side longissimus muscle was extracted and weighed. Samples of the left-side longissimus muscle were collected on male fetuses and immediately frozen in liquid nitrogen before storing at −80°C. Another group of ewes (*n* = 10) went to term. Wether lambs (*n* = 3) were weighed prior to a longissimus muscle biopsy being performed using a punch biopsy on 42-d (left side at 12th rib; PW1) and 65-d (right side at 12th rib; PW2) of age prior to weaning at d 75. Wether (*n* = 3) lambs that were biopsied at 42 and 65 d of age were finished on forages to 243-d of age (MAT) and slaughtered at the Clemson University Meat Lab. A live weight was collected prior to transport and longissimus muscle samples were collected from the left side at the 13th rib at slaughter. Longissimus muscle samples were snap frozen in liquid nitrogen and stored at −80°C for subsequent RNA extraction. After slaughter, carcasses were allowed to chill overnight at 4°C and then the right-side longissimus muscle was excised and weighed.

### Sample preparation for miRNA sequencing

Total RNA was extracted from longissimus muscle tissue using Trizol reagent (Invitrogen, Thermo Fisher Scientific, Waltham, MA) according to the manufacturer. Any genomic DNA contamination was removed from the RNA with a DNA-free Kit (Ambion, Carlsbad, CA) according to the manufacturer. A Nanodrop 1 spectrophotometer (Thermo Fisher) was used to quantify total RNA. RNA integrity numbers (RIN) were generated using an Agilent 2,100 Bioanalyzer (Agilent Technologies, Santa Clara, CA) according to the manufacturer and all RIN values were above 7. Total RNA samples were stored at −80°C until being shipped on dry ice to Novogene (Durham, NC) for library preparation and sequencing.

### Library preparation and sequencing data analysis

Three μg of total RNA per sample was used to construct a small RNA library and index codes were added using NEBNext^®^ Multiplex Small RNA Library Prep Set for Illumina^®^ (NEB, United States) according to the manufacturer’s recommendations. Library quality was evaluated with an Agilent Bioanalyzer 2,100 using DNA High Sensitivity Chips. Clustering of index-coded samples was done using a cBot Cluster Generation System and a TruSeq SR Cluster Kit v3-cBot-HS (Illumina) according to the manufacturer’s instructions. Library preparations were sequenced using an Illumina platform and 50bp single-end reads were generated.

Bowtie was used to map reads to the reference genome (Langmead et al., 2009). miRBase20.0 was used as a reference and known miRNA were identified with mirdeep2 (Friedländer et al., 2012) and srna-tools-cli. Reads from protein-coding genes, repeat sequences, rRNA, tRNA, snRNA, and snoRNA were removed with RepeatMasker. Unmapped reads were predicted using miREvo (Wen et al., 2012) and mirdeep2 based on characteristics of a hairpin structure: the secondary structure, Dicer cleavage site and minimum free energy of the small RNA. miRNA expression was estimated by transcripts per million and normalized (Zhou et al., 2010). Prediction of target genes of miRNA was performed by miRanda (Enright et al., 2003). Gene Ontology (GO) enrichment analysis was performed on target mRNA of differentially expressed miRNAs using GOseq based Wallenius non-central hyper-geometric distribution (Young et al., 2010) to adjust for gene length bias. Target gene candidates were enriched in KEGG pathways using KOBAS software (Mao et al., 2005; Kanehisa et al., 2008).

### Muscle fiber histology

At harvest, longissimus muscle samples at the 12/13th rib were collected, placed in a form with optimal cutting temperature compound (OCT; ThermoFisher), and immediately frozen in liquid nitrogen. Muscle samples were stored at −80°C until subsequent muscle histology was performed. Muscle samples were cryosectioned at a thickness of 10 μm, fixed for 2 min in ice cold acetone, and stained according to Greene et al. (2019) with modifications. Two tissue sections per animal were used for Type I/II and Type IIa/IIx myofiber typing. Cryosections of muscle samples were stained to determine Type I and Type II myofibers using primary antibodies (MHC-fast mouse IgG1, My-32, Abcam, ab51263, RRID:AB_2297993; MHC-slow mouse IgG2b, Developmental Studies Hybridoma Bank [DHSB], BA-F8, RRID:AB_10572253) and secondary antibodies (Alex Fluor 546 goat anti-mouse IgG1, Thermo Fisher, A-21123, RRID:AB_2535765) and Alexa Fluor 488 goat anti-mouse IgG2b, Thermo Fisher, A-21141, RRID:AB_2535778). Additional cryosections were stained to determine Type IIa or Type IIx myofibers using primary antibodies (MHC-Type IIa mouse IgG1, DSHB, SC-71, RRID:AB_2147165; MHC-Type IIx mouse IgM, DHSB, 6H1, RRID:AB_1157897) and secondary antibodies (Alex Fluor 546 goat anti-mouse IgG1, Thermo Fisher, A-21123, RRID:AB_2535765; Alex Fluor 488 goat anti-mouse IgM, Thermo Fisher, A-21042, RRID:AB_2535711). Sections were also counterstained with Alexa Fluor 633 wheat germ agglutinin at 10 μg/ml (Invitrogen, W21404) to outline muscle fiber membranes (Kostrominova, 2011). Stained muscle sections were mounted in Prolong Gold (P36939, Invitrogen) and samples were imaged using a Leica DMi8 widefield microscope system equipped with a Nikon ×20 objective (N.A. = 0.40) and a Leica DFC 9000 GTC Camera (Leica Microsystems, Buffalo Grove, IL). Camera exposure times were kept constant for all samples within each antibody group. At least ten unique sample regions (each measuring 670.15 µm × 670.15 µm) were imaged per section. To image samples stained with Alex Fluor 488 (depicted in green), we used a GFP filter cube (Ex/Em 455-495/505-555 nm). A Cherry filter (Ex/Em 540-580/592-668 nm) was used to image samples stained with Alexa Fluor 546 (depicted in red). For Alexa Fluor 633 (depicted in magenta), a Y5 filter cube (Ex/Em 600-660/662-738 nm) was used for imaging. Images were collected and exported as. TIF for analyses using Leica LAS-X software (version 3.6.0.20104, Leica Microsystems). Number and cross-sectional area of Type I/II myofibers and Type IIa/IIx myofibers were counted on four images per animal using ImageJ software (NIH, https://imagej.nih.gov/ij/docs/guide/146-1.html) by a single trained individual. Results were averaged by image and subjected to statistical analyses as described below.

### *In vitro* cell culture

Satellite cells were isolated according to Li et al. 2009 and Danoviz and Yablonka-Reuveni (2012). In brief, muscle tissue was be stripped of connective tissue and any fat, then further processed with a sterile food processor. The tissue was then enzymatically digested for 60 min in a 37°C water bath with 1.5 mg/ml pronase in PBS to facilitate satellite cell liberation. Tissue slurry was shaken vigorously every 10 min during the digestion. Following digestion, the tissue slurry was centrifuged at 800 × g for 10 min to separate the digested tissue from the pronase, which was discarded as supernatant. From this point the tissue slurry was resuspended in PBS 1:1 with the volume of the tissue pellet. Then the tissue slurry was triturated until sufficiently homogenized to liberate the satellite cells. The tube was then centrifuged at 400 × g for 10 min. Supernatant was decanted and retained. This process was completed 2 times to allow for complete liberation of satellite cells from the basal lamina. Following the 2^nd^ centrifugation the supernatant was filtered through a 250 µm and a 70 μm cell strainer and cells were pelleted at 800 x g for 10 min. Cells were resuspended for 2 h to allow for debris removal in a preplating media [Dulbecco’s modified eagle’s medium (DMEM; Gibco, Thermo Fisher) + 10% fetal bovine serum (FBS; Avantor, VWR, Radnor, PA) + 1% penicillin/streptomycin (Corning, VWR)]. After 2 h, cells were transferred to a new flask for selective adhesion to increase satellite cell population purity for an additional 24 h (Gharaibeh et al., 2008). Cells were pelleted following the preplating, counted with a hemocytometer, and frozen (DMEM + 20% FBS + 10% DMSO) for later experiments.

Satellite cells were cultured to assess the impact of miR-29a inhibition by AntogmiR-29a (Creative Biogene, Shirley, NY) during the proliferation or differentiation stages of cell development. Experiments were run in duplicate. Satellite cells were plated in 0.1% gelatin coated 24 well plates at 20,000 cells/well and 0.1% gelatin coated 96 well plates at 5,000 cells/well for imaging. Cultures were seeded and incubated for 48 h to reach ∼60% confluence prior to AntagomiR-29a transfection. Following treatment, cultures were grown for 4 d (the proliferation phase) in growth media [DMEM high glucose, 20% FBS, 1% penicillin/streptomycin, and 0.1% gentamicin (VWR)]. Cultures were differentiated for 4 d (the differentiation phase) in differentiation media (DMEM low glucose, 2% FBS, 1% penicillin/streptomycin, and 0.1% gentamicin). Cell media was changed every 2 d.

### Cell transfection

Satellite cell cultures were transfected using RNAiMAX lipofectamine (ThermoFisher) according to manufacturer recommendations. AntagomiR-29a was synthesized by Creative Biogene and administered at two levels 100 and 300 nM. Cells were transfected either on d 0 of the proliferation phase or on d 0 of the differentiation phase (d 4 of proliferation). Lipofectamine without miRNA was used as a control in addition to cells grown with no manipulation (Control). Cultures were collected for RNA extraction using Trizol at d 1 and 4 of growth and d 4 of differentiation.

### miRNA PCR

miRbase was used to obtain miRNA sequences for *Ovis aries* and then miRNA sequences were matched in the TaqMan assay database (ThermoFisher). miRNA was converted to cDNA with the TaqMan miRNA reverse transcription kit (ThermoFisher). TaqMan Small RNA Assay kits (ThermoFisher) were used for miR-29a (assay no. 000412; catalog no. 4427975), miR-22-3p (assay no. 242214_mat; catalog no. 444886), miR-133 (assay no. 000458; catalog no. 4427975), miR-127 (assay no. 008411_mat; catalog no. 4440886), miR-299-5p (assay no. 000600; catalog no. 4427975), miR-1 (assay no. 000385; catalog no. 4427975), and miR-206 (assay no. 000510; catalog no. 4427975). The housekeeping gene U6 snRNA was selected and the U6 snRNA TaqMan Assay Kit (assay no. 001973; catalog no. 4427975; ThermoFisher) was used for normalization of miRNA gene expression for tissue samples. The Taqman PCR assay kit and a Quant Studio3 Real-Time PCR system were used for qPCR according to the manufacturer instructions.

### Hoechst DNA assay

Cell culture proliferation was assessed using the procedures of Velleman et al. (2019) with modifications. The DNA content of samples was measured using Hoechst 33,342 fluorochrome (ThermoFisher). Cells were harvested daily starting on d 1 of proliferation with Trypsin-EDTA (0.25%) and stored at −80°C until assay. Cells were allowed to thaw on ice and then homogenized. The sample (100 μL) was combined with 100 μL of 0.2% (1 mg/ml) Hoechst dye in 0.1 M NaCl, 10 mM EDTA, 10 mM Tris with a pH 7.0. Plates were incubated in the dark for 10 min and fluorescence read at Ex; Em 330/80; 460/40 using a BioTek Synergy HT (Winooski, VT). A standard curve using purified double-stranded DNA was used to determine sample DNA concentrations. Experiments were conducted in duplicate with 3 replicate wells per sample. The intra-assay variance was < 9.6% and the inter-assay variance was 9.5%.

### Cell culture immunofluorescence

The number of differentiated nuclei was assessed by antibody staining of myogenin (MYOG) positive nuclei. The expression of MYOG can be used as a marker for differentiated myoblast cells (Schmidt et al., 2019). Media was removed after 4 d of differentiation and cells were fixed with 4% paraformaldehyde at room temperature for 15 min. Cells were then permeabilized with 0.1% TritonX-100 for 10 min at room temperature and then blocked with a 3% BSA solution in PBS for 60 min at room temperature. Plates were incubated with a MYOG primary antibody 1:10 in PBS (Myogenin mouse IgG1, DHSB catalog no. F5D, RRID: AB_2146602) overnight at 4°C. Following incubation, plates were allowed to come to room temperature for 30 min. Primary antibody was removed with thorough washing and plates were incubated with a 1:1,000 Texas Red secondary antibody (goat anti-mouse IgG1, ThermoFisher catalog no. A-10530, RRID:AB_2534035) for 60 min protected from light at room temperature. Unbound secondary antibody was removed with thorough washing. Total nuclei were stained with Hoechst 33,342 (10 μg/ml, ThermoFisher) for 10 min protected from light at room temperature. Excess Hoechst dye was removed, and cells were imaged immediately. Twelve unique sample regions were imaged per well using a Cytation1 (BioTek) with a × 10 objective and a DAPI filer cube (DAPI, blue, EX; Em 377/50; 447/60) and a Texas Red filter cube (Texas Red, red, Ex; Em 586/15; 647/57). A single trained individual then counted the total nuclei and the total nuclei expressing MYOG. Results are expressed as the percentage of the total nuclei expressing MYOG.

### Statistical analysis

Analysis of variance (ANOVA) followed by Fisher’s Protected Least Significant Difference Test (FPLSD) was used to determine the effect of animal age (gd85, gd110, gd133, d42, d65 and d243) on lamb body weight, longissimus muscle mass, and qPCR. ANOVA and FPLSD were also used for muscle fiber histology cross-sectional area data, but the assumption of normality required was not met for these data. The data were averaged by image and several different distributions were used to perform the analysis. Fortunately, the different distributions all yielded similar results, and therefore results are presented in original scale for ease of interpretation. ANOVA was used to determine the effects of treatment and experiment on cell culture data, followed by *a priori* contrasts to compare treatment groups to the control. The Generalized Linear Mixed Model (GLIMIX) and General Linear Model (GLM) procedures of SAS (Version 9.4) of SAS 9.4 (SAS Institute) for the ANOVA, FPLSD, and contrast calculations. *p*-values less than 0.05 were considered evidence of statistical significance.

Comparison of miRNA expression analysis was conducted using the DESeq R package (1.8.3) and the Benjamini and Hochberg method was used to adjust *p* values.

## Results

### Lamb characteristics

Body weight increased (*p* < 0.0001) at each timepoint of development examined in this study (Table 1). Body weight mass increased by 113-fold from 0.5 kg at PN1 to 57 kg at MAT. Longissimus thoracis et lumborum (LM) weight increased (*p* < 0.0001) from PN1 to MAT by 139-fold. The weight of the LM was greater (*p* < 0.05) at PN3 compared to PN1 with PN2 being intermediate. The lambs were grown to near maturity, plateau of longissimus thoracis et lumborum muscles (MAT), which represents postweaning growth period.

**TABLE 1:** Changes in body weight (BW), longissimus thoracis et lumborum (LM) weight, muscle fiber type and cross-sectional area by developmental time point.

	PN1 gd85	PN2 gd110	PN3 gd133	PW1 d42	PW2 d65	MAT d243	SEM
BW, kg	0.471^a^	2.28^b^	3.912^c^	19.566^d^	28.652^e^	56.90^f^	0.376
LM, g	6.30^a^	31.09^ab^	42.33^b^	—	—	884.15^c^	11.56
Muscle fiber type, %							
Type I	8.76^b^	8.71^b^	17.07^a^	—	—	7.81^b^	0.98
Type II	91.24^a^	91.29^a^	82.93^b^	—	—	92.19^a^	0.98
Type IIa	86.16^a^	81.12^a^	77.23^a^	—	—	30.68^b^	4.85
Type IIax	0^c^	9.59^bc^	13.98^b^	—	—	59.75^a^	4.37
Type IIx	13.84	8.29	8.78	—	—	9.57	1.55
Muscle fiber cross-sectional area, µm^2^							
Type I	87.80^d^	144.26^c^	254.29^b^	—	—	2,429.77^a^	
Type II	70.76^d^	173.36^c^	263.54^b^	—	—	2,528.13^a^	
Type IIa	60.08^d^	117.18^c^	215.04^b^	—	—	1820.30^a^	
Type IIax		164.54^b^	397.84^b^	—	—	2,998.44^a^	
Type IIx	68.61^c^	137.25^c^	277.84^b^	—	—	3,185.46^a^	

^abcdef^Means in the same row with uncommon superscripts differ (*p* < 0.05).

### Muscle fiber histology

**FIGURE 1:**
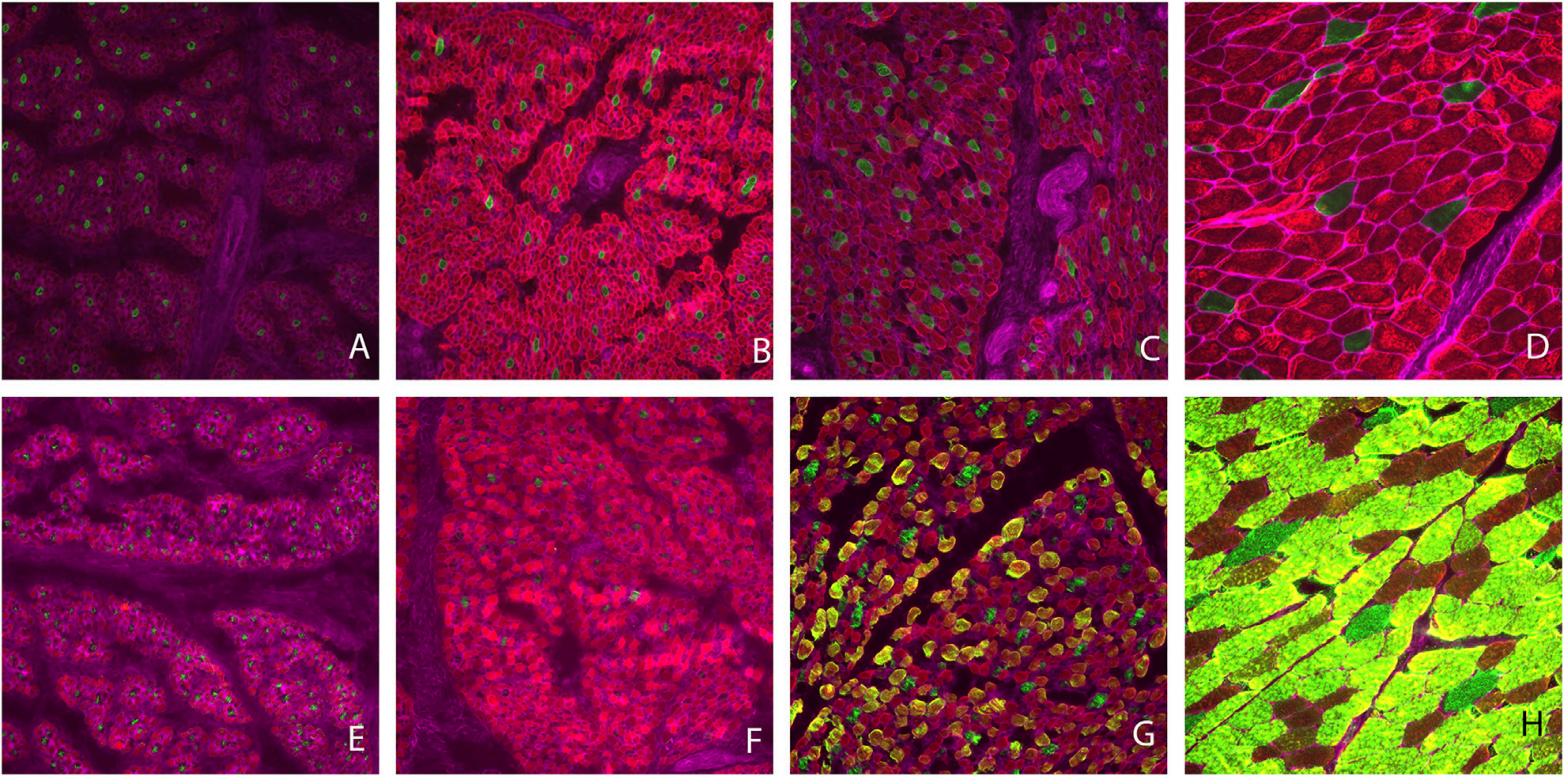
Muscle fiber cross sectional area images. Type I fibers are stained green and Type II fibers are red **(A–D)**. Type IIa fibers are stained red, Type IIx fibers are stained green and fibers expressing Type IIa and IIx proteins (Type IIax) are yellow in color **(E–H)**. Gestational d 85 samples **(A,E)**, gestational d 110 **(B,F)**, gestational d 133 **(C,G)**, maturity d 243 postnatal **(D,H)**.

Muscle fiber cross sectional images with Type I and II staining or Type IIa, IIx, and IIax are shown in Figure 1. Muscle fiber cross-sectional of Type I myofibers was greater (*p* < 0.001) at each timepoint during development. Overall hypertrophy of Type I myofibers increased from 88 μm^2^ at PN1 to 2,430 μm^2^ at MAT. Type I fibers represented about 8.43% of total myofibers at PN1, PN2, and MAT; however, type I myofiber percentage was greater (*p* < 0.0001) at PN3, 17.07%, compared to all other developmental timepoints. At each prenatal developmental stage, cross-sectional area increased by 64% at PN2 and by 76% at PN3, demonstrating that muscle fiber hypertrophy is on-going during the period of rapid fetal growth in the last trimester of gestation. Type II fiber area increased (*p* < 0.0001) at each development stage. Cross-sectional area of Type II fibers increased from 71 μm^2^ at PN1 to 2,528 μm^2^ at MAT. Type II fibers were further classified as Type IIa, IIx, and IIax (transitioning). Type IIa fiber size increased (*p* < 0.0001) at each development point evaluated from 60 μm^2^ at PN1 to 1820 μm^2^ at MAT. As a percentage of the total fibers, Type IIa fiber number was decreased (*p* < 0.0001) for the MAT developmental time point compared to all other times. Type IIx fibers size increased (*p* < 0.0001) as development progressed from prenatal to postnatal stage. Type IIx cross-sectional area did not differ between PN1 and PN2, however fiber size increased from PN2 to PN3 and PN3 to MAT. Type IIx fiber area increased from 69 μm^2^ at PN1 to 3,185 μm^2^ at MAT. Type IIx fibers as a percentage of the total fibers did not differ by developmental timepoint. Fibers that expressed both IIa and IIx proteins were classified as transitional fibers (IIax). No transitional fibers were found in PN1 samples. Fiber area of Type IIax fibers increased (*p* < 0.01) as development progressed. PN2 samples did not differ from PN3 fibers but cross-sectional area increased from PN3 to MAT. Type IIax fiber area increased from 164 μm^2^ at PN2 to 2,998 μm^2^ MAT. The percentage of Type IIax myofibers was similar during the prenatal stage (PN2 vs. PN3) but lower than MAT. The percentage of type IIa and IIax myofibers at MAT differed from the prenatal stage when Type IIax myofibers were non-existent or in low abundance.

### miRNA sequencing

**FIGURE 2:**
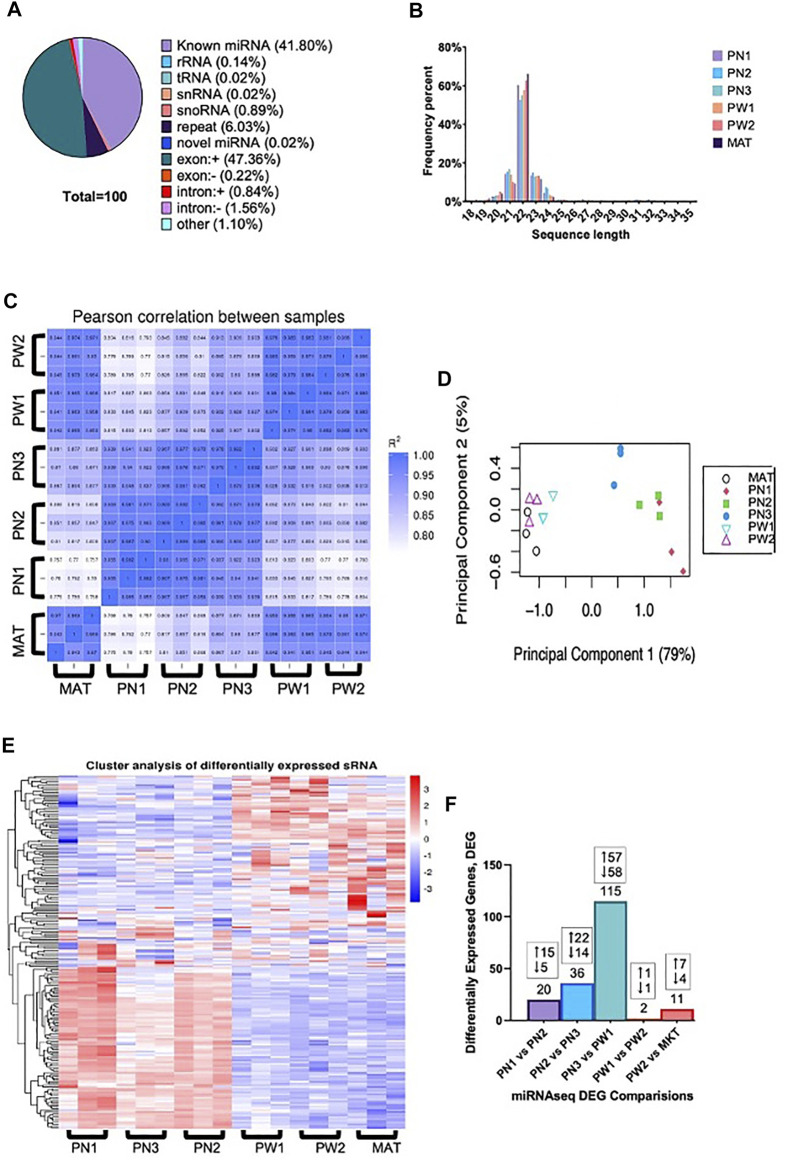
Small RNA analysis of ovine skeletal muscle at 6 developmental time points. **(A)** The classification of small RNA reads. **(B)** Distribution of small RNA sequence lengths from six developmental time points. **(C)** Correlation heat map of individual samples within developmental timepoints. **(D)** Principal component analysis of individual samples. **(E)** Hierarchical clustering analysis of individual samples within each developmental timepoint. **(F)** Number of differentially expressed miRNA from each comparison made with the number of up and downregulated miRNA denoted.

The total raw reads generated by sequencing was 396,602,151 with a minimum of 19,219,284 reads per individual sample and all samples had a Q30 of >97.00%. Reads containing >10% N (<0.000%), 5′ primer contaminants (0.005%), and/or did not contain the 3′ primer and the insert tag (0.842%) were excluded from the data. The 3′ primer sequence was trimmed and reads containing poly A/T/G/C (0.028%) were excluded. The remaining reads (99.125%) were used for mapping. Annotated reads were classified as known miRNA (41.80%), rRNA (0.14%), tRNA (0.02%), snRNA (0.02%), snoRNA (0.89%), repeat (6.03%), novel miRNA (0.02%; Supplementary Table S1), exon (±, 47.58%), intron (±, 2.40%), and other (1.10%) (Figure 2A). The largest portion was reads that mapped to exon regions with known miRNA being the second largest percentage. Reads length were also obtained and most reads ranged from 20–24 nt, with 22 nt being the frequency (Figure 2B). There was a high correlation between samples of the same developmental time point, indicating the reliability between samples (Figure 2C). Additionally, principal component analysis showed that developmental order could be captured, and a clear distinction was present between prenatal and postnatal samples (Figure 2D). This difference between pre and postnatal samples was also seen in the differentially expressed miRNA (Figure 2E).

**FIGURE 3:**
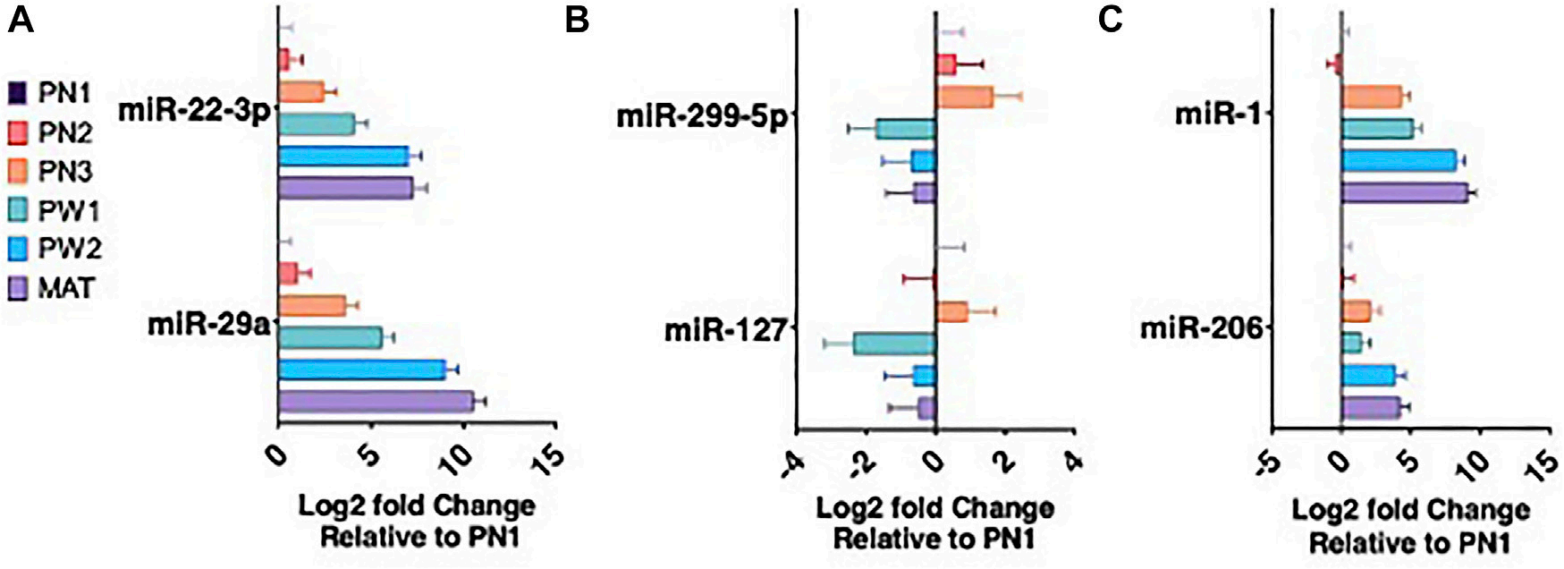
miRNA expression during six developmental time points. miRNA upregulated during development **(A)**. miRNA downregulated during development **(B)**. Muscle specific miRNA expression **(C)**.

There were 142 unique miRNA differentially expressed (*P*_*adj*_ < 0.05) between the five stages of longissimus muscle hypertrophy examined in this study (Figure 2F). No miRNA were differentially expressed at every growth stage examined but two miRNAs, miR-29a and miR-431, were differentially expressed in four comparisons. miR-29a was continuously upregulated from PN2 to MAT. miR-431 was down regulated from PN1 to PW1 and PW2 to MAT; however, miR-431 was not differentially expressed between PW1 and PW2. For validation of sequencing, miR-29a, -22-3p, -299-5p, and 127 were chosen due to their expression over several developmental timepoints and high or low abundance, respectively (Figures 3A,B). Expression of mir-29a and -22-3p increased (*p* < 0.05) during development. Overall, the increasing log2 fold change for miR-29a and -22-3p agreed with the sequencing results that showed upregulation during development (Figure 3A). miR-299-5p and -127 decreased (*p* < 0.05) in expression during postnatal development and this agreed with sequencing results (Figure 3B). Additional miRNA known to be present in muscle but not annotated in sequencing results, miR-1 and -206, were examined using qPCR (Figure 3C). miR-1 and -206 were both present at all developmental time points and expression increased (*p* < 0.05) as development progressed.

During the mid-prenatal stage (PN1 vs. PN2), 20 miRNA were differentially expressed (*P*_*adj*_ < 0.05) in this comparison (Table 2). miR-150 was up-regulated by 2.1-fold (*P*_*adj*_ < 0.0001) and miR-154b-3p was down-regulated by -2.4-fold (*P*_*adj*_ < 0.0001). Fourteen other miRNA were down-regulated (*P*_*adj*_ < 0.05) and four other miRNA were up-regulated (*p* < 0.05) from PN1 to PN2. Of these downregulated miRNA, miR-376, miR-17-5p and miR-431 have documented roles in skeletal muscle of the neonate and appear to alter satellite cell differentiation.

**TABLE 2:** Differentially expressed miRNA during mid prenatal phase (PN1 = gd85; PN2 = gd110) by significance level.

miRNA	PN1 TPM	PN2 TPM	log2FoldChange	*P*_*adj*_ *.*
oar-miR-150	91.79	485.40	2.1168	4.75E-08
oar-miR-154b-3p	104.76	13.03	−2.3920	8.88E-07
oar-miR-3959-3p	2,180.08	578.15	−1.6516	2.81E-04
oar-miR-487a-3p	140.56	37.32	−1.5813	3.04E-03
oar-miR-376d	402.22	152.21	−1.2468	5.96E-03
oar-miR-134-5p	741.58	351.42	−1.0009	6.12E-03
novel_159	1.40	14.19	1.7999	1.27E-02
oar-miR-665-3p	408.60	170.16	−1.1023	3.78E-02
oar-miR-496-5p	13.29	2.36	−1.4995	4.32E-02
oar-miR-1185-5p	71.02	25.14	−1.2216	4.32E-02
oar-miR-154a-3p	7,426.57	3,239.99	−1.0434	4.32E-02
novel_61	9.95	39.78	1.4153	4.32E-02
novel_7	2.25	12.75	1.5428	4.32E-02
oar-miR-376a-3p	526.98	197.21	−1.1641	4.39E-02
oar-miR-376e-3p	717.47	270.19	−1.1568	4.39E-02
oar-miR-17-5p	723.12	318.25	−1.0304	4.39E-02
oar-miR-431	7,283.58	2,116.71	−1.315	4.39E-02
novel_99	6.70	29.79	1.4065	4.57E-02
oar-miR-410-5p	52.07	17.95	−1.1921	4.98E-02
oar-miR-485-3p	1,178.44	592.23	−0.8902	4.98E-02

During the late prenatal stage of development (PN2 vs. PN3), 36 miRNA were differentially expressed (*P*_*adj*_ < 0.05) for this comparison (Table 3). miR-665-3p was down-regulated by -2.2-fold (*P*_*adj*_ < 0.0001) and novel_32 was up-regulated by 1.9- fold (*P*_*adj*_ < 0.0001). Thirteen additional miRNA were up-regulated (*P*_*adj*_ < 0.05) and 21 were down-regulated (*P*_*adj*_ < 0.05) during the late prenatal stage (PN2 vs. PN3). miR-133 and miR-143 were up-regulated and are known myomiRs with involvement in skeletal muscle hypertrophy.

**TABLE 3:** Differentially expressed miRNA during mid prenatal phase (PN2 = gd110; PN3 = gd133) by significance level.

miRNA	PN2 TPM	PN3 TPM	log2FoldChange	*P*_*adj*_ *.*
oar-miR-665-3p	105.53	19.76	−2.1548	7.42E-07
oar-miR-136	4,205.65	12,564.74	1.4807	1.92E-05
oar-let-7f	232,325.25	497,336.61	1.0648	1.92E-05
oar-miR-758-3p	2,148.68	718.67	−1.4824	1.92E-05
oar-miR-376a-3p	122.33	31.74	−1.7540	1.92E-05
novel_32	26.48	121.52	1.9340	3.46E-05
oar-miR-154a-3p	2013.45	616.16	−1.5747	3.46E-05
oar-miR-431	1,318.53	327.11	−1.7979	3.46E-05
oar-miR-133	44,300.34	97,376.40	1.0930	5.05E-05
oar-miR-134-5p	217.18	88.23	−1.2304	5.05E-05
oar-miR-376d	94.20	30.84	−1.4645	2.30E-04
oar-miR-3959-3p	357.75	106.33	−1.5693	2.93E-04
oar-miR-1185-3p	712.52	113.66	−2.0861	3.55E-04
oar-miR-485-3p	365.87	128.05	−1.3896	3.55E-04
oar-miR-541-5p	685.94	351.68	−0.9286	5.67E-04
oar-miR-411a-3p	9,364.18	4,496.96	−1.0081	1.10E-03
oar-miR-29a	92.88	245.24	1.2835	1.71E-03
oar-miR-3957-5p	241.13	100.94	−1.1536	4.46E-03
oar-miR-376b-3p	254.12	83.64	−1.4026	4.55E-03
novel_91	5.83	32.93	1.8187	5.79E-03
oar-miR-143	1,154,220.57	2,416,323.96	0.9992	6.21E-03
oar-miR-541-3p	27.80	7.33	−1.5770	6.24E-03
novel_169	2.00	13.50	1.8854	6.32E-03
oar-miR-539-3p	147.10	61.36	−1.1402	9.63E-03
oar-let-7a	50,937.12	101,393.48	0.9319	1.09E-02
oar-miR-494-3p	4,633.28	2,740.92	−0.7292	1.15E-02
oar-let-7g	76,122.52	120,328.58	0.6411	1.30E-02
oar-miR-411b-3p	40.33	13.62	−1.3109	2.56E-02
novel_89	13.79	37.62	1.2308	2.67E-02
oar-miR-127	288,665.48	195,115.94	−0.5504	2.87E-02
oar-miR-412-3p	6,121.91	11,761.58	0.8756	3.03E-02
oar-miR-487a-3p	23.14	5.21	−1.5171	3.16E-02
oar-miR-410-5p	11.10	1.78	−1.5813	4.05E-02
oar-miR-376c-5p	887.36	1,509.02	0.7259	4.11E-02
novel_15	0.73	6.39	1.5986	4.57E-02
oar-miR-370-3p	87,715.07	34,345.31	−1.1449	4.82E-02

During the transition from prenatal to postnatal development (PN3 vs. PW1), this stage had the most differentially expressed miRNA with 115 (Table 4). The largest magnitude (>3) log fold changes all came from the PN3 vs. PW1 comparison, miR-22-3p was up-regulated by 4.5-fold (*P*_*adj*_ < 0.0001) and novel_91 was down-regulated by -3.7-fold (*P*_*adj*_ < 0.0001). An additional 11 miRNA had a fold-change greater than 3, 7 were up-regulated and 4 were down regulated. Fifty-two miRNA were down-regulated (*P*_*adj*_ < 0.05) and 50 were up-regulated (*P*_*adj*_ < 0.05) with a fold-change < 3.

**TABLE 4:** Differentially expressed miRNA during prenatal to postnatal transition phase (PN3 = gd133; PW1 = pd42) by significance level.

miRNA	PN3 TPM	PW1 TPM	log2FoldChange	*P*_*adj*_ *.*
oar-miR-22-3p	3,253.46	80,720.92	4.525	1.04E-57
oar-miR-299-5p	1,130.39	163.00	−2.753	3.26E-37
oar-miR-487b-3p	1,586.25	382.71	−2.0275	3.04E-24
oar-let-7g	55,592.12	134,263.09	1.2647	8.64E-18
oar-miR-30c	2,888.27	17,727.15	2.5525	2.01E-17
oar-miR-127	90,359.34	36,459.82	−1.3007	8.43E-17
oar-miR-432	4,637.76	964.83	−2.2213	1.17E-16
oar-miR-30d	58,769.57	287,068.30	2.2413	2.04E-16
oar-miR-29a	114.76	892.44	2.8509	2.86E-15
oar-miR-143	1,115,871.88	2,978,500.86	1.4042	2.94E-15
oar-miR-299-3p	365.94	73.79	−2.243	3.23E-14
oar-miR-381-3p	1,114,970.39	198,832.88	−2.417	3.64E-14
oar-miR-410-3p	1,324.49	146.52	−3.0324	6.13E-14
oar-miR-154a-3p	282.06	29.47	−3.1168	6.26E-14
oar-miR-3959-5p	2,487.10	760.65	−1.6855	3.08E-13
novel_101	2.42	56.15	4.0619	5.49E-13
oar-miR-376c-5p	702.86	105.15	−2.6425	6.86E-13
oar-miR-431	150.00	17.17	−3.0003	3.11E-12
oar-miR-16b	396.33	3,176.06	2.858	3.79E-12
oar-miR-495-3p	14,909.28	2,664.33	−2.3976	1.10E-11
oar-miR-493-5p	25,857.82	3,413.60	−2.7822	1.12E-11
oar-miR-27a	732.01	2,872.37	1.9279	1.35E-11
oar-miR-655-3p	5,093.71	575.75	−2.9609	7.15E-11
oar-miR-133	45,326.48	198,971.38	2.0734	1.01E-10
oar-miR-136	5,808.63	1,047.80	−2.3698	5.99E-10
novel_7	5.81	66.85	3.2284	8.29E-10
oar-miR-329b-3p	1,209.13	269.32	−2.088	3.31E-09
oar-miR-25	4,944.24	18,970.32	1.8852	4.35E-09
oar-miR-541-5p	162.78	45.44	−1.8002	5.87E-09
oar-miR-382-5p	5,232.22	1,504.11	−1.753	7.44E-09
oar-miR-191	3,644.91	11,054.01	1.5674	1.56E-08
oar-miR-150	351.46	2,376.73	2.592	1.86E-08
novel_159	3.66	39.97	3.14	4.37E-08
oar-miR-10b	295,096.57	791,364.41	1.3979	5.36E-08
oar-miR-103	902.93	4,755.78	2.2692	2.31E-07
oar-miR-125b	5,988.66	11,625.02	0.94814	4.41E-07
oar-miR-194	90.40	394.65	2.0266	9.84E-07
oar-miR-3956-5p	4,762.74	424.20	−3.0799	9.84E-07
novel_87	0.00	14.90	4.2219	5.24E-06
oar-miR-21	171,273.72	356,873.16	1.0442	7.52E-06
novel_4	12.28	78.51	2.4509	7.56E-06
oar-miR-379-5p	134,717.62	74,976.28	−0.83758	1.05E-05
oar-miR-382-3p	3,434.37	1,334.07	−1.3301	1.54E-05
oar-miR-221	32.74	233.10	2.5456	1.95E-05
oar-miR-30a-3p	1,360.59	2,175.92	0.67301	1.99E-05
novel_13	0.82	23.95	3.5678	2.31E-05
oar-miR-26a	142,269.96	387,849.86	1.4038	3.16E-05
oar-miR-369-3p	3,205.77	599.33	−2.2194	5.26E-05
oar-miR-3958-5p	179.07	36.70	−2.1109	5.26E-05
oar-miR-3956-3p	58.10	6.98	−2.6236	5.75E-05
oar-miR-495-5p	113.37	29.91	−1.7905	6.71E-05
novel_91	14.99	0.00	−3.6814	7.28E-05
oar-miR-380-3p	2,225.71	710.98	−1.5783	7.35E-05
novel_67	49.85	139.43	1.4286	1.08E-04
oar-miR-381-5p	25.66	3.39	−2.4986	1.52E-04
oar-miR-3955-5p	835.09	394.68	−1.0563	1.55E-04
oar-miR-107	128.68	391.89	1.5352	1.71E-04
novel_84	0.00	11.01	3.5774	2.40E-04
oar-miR-1185-3p	52.11	16.42	−1.5919	2.69E-04
oar-miR-3958-3p	47,442.40	20,242.12	−1.1938	3.39E-04
oar-miR-411a-3p	2067.40	546.75	−1.7827	5.15E-04
oar-miR-494-3p	1,270.31	755.52	−0.7418	6.25E-04
oar-miR-3955-3p	16.99	0.92	−2.9307	6.89E-04
oar-miR-181a	6,692.89	14,436.41	1.0794	9.16E-04
oar-miR-23a	2,455.76	7,624.23	1.5412	9.92E-04
novel_122	17.19	49.42	1.4476	1.07E-03
oar-miR-200c	3.14	20.69	2.3002	1.39E-03
novel_128	5.78	21.22	1.7154	1.48E-03
novel_54	0.00	4.71	3.0279	2.59E-03
oar-miR-543-3p	2,899.34	483.46	−2.1936	2.59E-03
oar-miR-376c-3p	259.56	85.30	−1.5059	2.70E-03
oar-miR-1193-5p	9.14	0.44	−2.7133	3.12E-03
oar-miR-329a-3p	31.82	8.82	−1.6675	3.34E-03
oar-miR-30b	1,431.93	7,886.11	2.0949	3.92E-03
oar-miR-493-3p	17,054.16	7,651.77	−1.1124	4.41E-03
oar-miR-329b-5p	23.91	5.34	−1.8633	5.17E-03
oar-miR-487a-5p	21.59	5.67	−1.6787	5.66E-03
oar-miR-17-5p	100.41	340.30	1.6048	5.69E-03
oar-let-7c	16,034.49	10,760.04	−0.56963	5.69E-03
oar-miR-655-5p	14.21	1.71	−2.3672	5.82E-03
oar-miR-370-5p	59.48	26.40	−1.1376	6.61E-03
oar-miR-30a-5p	56,262.60	97,321.10	0.77484	6.72E-03
novel_96	2.78	13.04	1.9389	7.16E-03
oar-miR-376a-3p	14.86	2.63	−2.0776	7.16E-03
oar-miR-376d	14.29	2.58	−2.0523	7.16E-03
novel_6	8.36	56.60	2.2011	7.43E-03
oar-miR-106a	17.22	56.22	1.5485	8.16E-03
novel_71	0.00	3.39	2.6493	9.51E-03
novel_83	0.00	3.50	2.6147	1.07E-02
oar-miR-487b-5p	22.13	5.47	−1.6954	1.09E-02
novel_61	13.52	42.26	1.483	1.10E-02
novel_42	0.20	4.84	2.5102	1.20E-02
oar-miR-412-3p	5,378.49	2,797.79	−0.91164	1.27E-02
oar-miR-323a-3p	917.06	352.11	−1.2845	1.33E-02
novel_8	7.16	33.85	1.867	1.40E-02
novel_75	9.16	27.85	1.4578	1.43E-02
oar-let-7f	229,818.32	311,018.30	0.43319	1.48E-02
oar-miR-26b	7,723.60	15,612.67	0.97488	1.48E-02
novel_5	37.21	89.02	1.186	1.50E-02
oar-miR-433-3p	486.83	246.10	−0.94394	1.50E-02
oar-miR-19b	909.29	1,537.82	0.74053	1.56E-02
oar-miR-412-5p	69.82	24.92	−1.3518	1.71E-02
novel_127	3.75	15.66	1.7598	1.77E-02
oar-miR-380-5p	95.19	49.81	−0.90109	2.05E-02
oar-miR-154b-5p	2,845.81	1,425.43	−0.95264	2.43E-02
novel_3	33.96	15.98	−1.0468	2.45E-02
oar-miR-409-5p	1,289.73	729.15	−0.79479	2.90E-02
novel_157	1.68	10.52	1.9707	2.92E-02
novel_52	0.20	4.45	2.2066	3.13E-02
oar-miR-10a	38,127.81	55,714.68	0.53872	3.50E-02
oar-miR-23b	3,386.13	6,868.51	0.96512	3.98E-02
novel_35	0.79	8.09	2.0227	4.54E-02
oar-miR-323b	63.49	13.28	−1.7337	4.57E-02
oar-miR-377-5p	27.26	9.33	−1.3625	4.99E-02
novel_85	0.00	2.71	2.0249	5.00E-02

During the postnatal phase (PW1 vs. PW2), this stage had the smallest number of differentially expressed (*P*_*adj*_ < 0.05) miRNA with just two, miR-29a (up-regulated 0.8 fold) and miR-127 (down-regulated -0.6 fold), and this may be due to the short time duration between sampling (∼25 days; Table 5). As longissimus muscle growth plateaus near maturity (PW2 vs. MAT), 11 miRNA were differentially expressed (Table 5). miR-431 was up-regulated 2.3-fold and novel_13 was down-regulated -1.9-fold for the PW2 vs. MAT comparison.

**TABLE 5:** Differentially expressed miRNA during postnatal phase of development (PW1 = pd42; PW2 = pd60) and postweaning phase of development (PW2 = pd60; MAT = pd 240) by significance level.

miRNA	PW1 TPM	PW2 TPM	log2FoldChange	*P*_*adj*_ *.*
oar-miR-29a	435.77	814.59	0.82216	3.03E-02
oar-miR-127	17,782.67	11,042.48	−0.64633	3.10E-02

miRNA	PW2 TPM	MAT TPM	log2FoldChange	*Padj.*
oar-miR-29a	579.32	1954.82	1.6739	1.55E-09
oar-miR-29b	17.47	78.60	1.9496	1.42E-06
oar-miR-431	3.13	32.38	2.2537	1.45E-03
oar-miR-16b	1,405.30	478.61	−1.4019	2.00E-03
oar-miR-3959-5p	289.34	153.88	−0.86824	2.19E-02
oar-miR-412-3p	771.84	368.68	−0.98391	2.19E-02
oar-miR-411b-5p	197.62	81.17	−1.1539	2.19E-02
novel_13	14.96	0.88	−1.9055	2.19E-02
oar-miR-376e-5p	13.57	2.03	−1.7191	2.51E-02
oar-miR-543-3p	145.03	827.55	1.6478	3.81E-02
oar-miR-494-3p	248.53	118.80	−0.96596	4.73E-02

Target genes of differentially expressed miRNA were identified and the functional associations of genes were assessed with GOseq (Young et al., 2010). Between all 5 comparisons made, 195 terms were significantly enriched, 86 were from biological process, 42 were from cellular component, and 67 were from molecular function (Table 6; Supplementary Tables S2–S6). Fifty-nine terms were expressed in one comparison, 24 in 2 comparisons, 19 in 3 comparisons, 89 in 4 comparisons, and 4 in all 5 comparisons. The PN1 vs. PN2 comparison had 119 enriched terms, PN2 vs. PN3 had 121 enriched terms, PN3 vs. PW1 had 138 enriched terms, PW1 vs. PW2 had 4 enriched terms, and PW2 vs. MRT had 158 enriched terms.

**TABLE 6:** **Summary of enriched Gene Ontology terms from each comparison**.

GO Accession	Description	Term type	P_adj_
Enriched Terms PN1 v PN2			
GO:0003824	Catalytic activity	Molecular function	7.99E-13
GO:0008152	Metabolic process	Biological process	7.99E-13
GO:0005488	Binding	Molecular function	6.10E-10
GO:0016787	Hydrolase activity	Molecular function	6.07E-09
GO:0043167	Ion binding	Molecular function	1.02E-08
Enriched Terms PN2 v PN3			
GO:0003824	Catalytic activity	Molecular function	1.21E-16
GO:0008152	Metabolic process	Biological process	4.69E-14
GO:0016787	Hydrolase activity	Molecular function	1.73E-11
GO:0005488	Binding	Molecular function	1.54E-09
GO:0070011	Peptidase activity, acting on L-amino acid peptides	Molecular function	2.80E-09
Enriched Terms PN3 v PW1			
GO:0003824	Catalytic activity	Molecular function	2.07E-20
GO:0008152	Metabolic process	Biological process	1.36E-14
GO:0016787	Hydrolase activity	Molecular function	4.09E-12
GO:0005488	Binding	Molecular function	1.89E-10
GO:0008233	Peptidase activity	Molecular function	2.51E-09
Enriched Terms PW1 v PW2			
GO:0016787	Hydrolase activity	Molecular function	4.54E-03
GO:0008152	Metabolic process	Biological process	4.54E-03
GO:0003824	Catalytic activity	Molecular function	8.58E-03
GO:0005975	Carbohydrate metabolic process	Biological process	1.04E-02
Enriched Terms PW2 v MKT			
GO:0003824	Catalytic activity	Molecular function	2.28E-12
GO:0008152	Metabolic process	Biological process	1.03E-11
GO:0071704	Organic substance metabolic process	Biological process	2.95E-09
GO:0044238	Primary metabolic process	Biological process	1.25E-08
GO:0044237	Cellular metabolic process	Biological process	2.30E-07

Target genes of the of the 142 differentially expressed miRNA were annotated using the Kyoto Encyclopedia of Genes and Genomes to examine the miRNA relationship to cellular pathways (Table 7; Supplementary Tables S7–S11). A total of 35 pathways were enriched when all comparisons were examined. The PN1 vs. PN2 comparison had 16 enriched pathways, PN2 vs. PN3 had 17 enriched pathways, PN3 vs. PW1 had 21 enriched pathways, PW1 vs. PW2 had 2 enriched pathways, and PW2 vs. MKT had 21 enriched pathways. One pathway, Proteasome, was enriched in all 5 comparisons. Eight pathways were enriched in 4 comparisons, 5 in 3 comparisons, 4 in 2 comparisons, and 17 in only 1 comparison. The PN3 vs. PW1 comparison had enriched pathways with the highest rich factors (a ratio of genes enriched to total genes annotated in a pathway) when compared the same pathway in one or more of the 4 other comparisons; indicating that more genes were enriched in the PN3 vs. PW1 comparison when compared to the others.

**TABLE 7:** Summary of enriched KEGG pathways from each comparison.

ID	Description	*P*_*adj*_
Enriched Pathways PN1 v PN2		
oas05140	Leishmaniasis	1.39E-02
oas04145	Phagosome	2.21E-02
oas05150	*Staphylococcus aureus* infection	2.21E-02
oas04210	Apoptosis	2.21E-02
oas04141	Protein processing in endoplasmic reticulum	2.21E-02
Enriched Pathways PN2 v PN3		
oas04142	Lysosome	1.09E-03
oas04141	Protein processing in endoplasmic reticulum	2.04E-03
oas05140	Leishmaniasis	2.04E-03
oas01100	Metabolic pathways	2.04E-03
oas04145	Phagosome	2.57E-03
Enriched Pathways PN3 v PW1		
oas04145	Phagosome	1.58E-03
oas04141	Protein processing in endoplasmic reticulum	3.10E-03
oas05140	Leishmaniasis	3.10E-03
oas05150	*Staphylococcus aureus* infection	3.10E-03
oas04976	Bile secretion	3.10E-03
Enriched Pathways PW1 v PW2		
oas04974	Protein digestion and absorption	4.15E-03
oas03050	Proteasome	2.31E-02
Enriched Pathways PW2 v MKT		
oas05140	Leishmaniasis	1.33E-03
oas05150	*Staphylococcus aureus* infection	2.24E-03
oas04152	AMPK signaling pathway	2.24E-02
oas05330	Allograft rejection	8.63E-02
oas04141	Protein processing in endoplasmic reticulum	1.17E-02

### Cell culture

**FIGURE 4:**
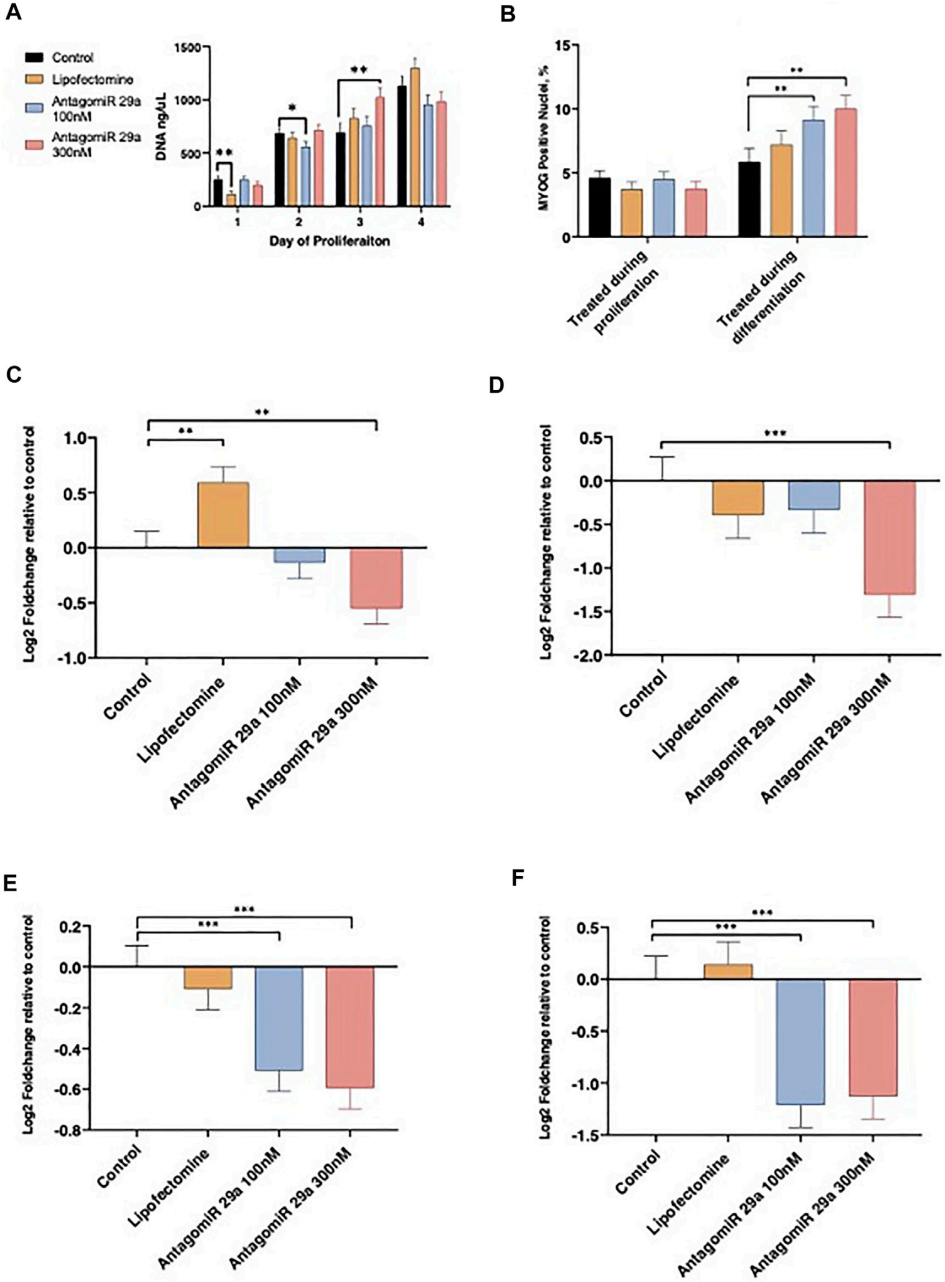
The effects of miR-29a inhibition on satellite cell proliferation, differentiation, and miRNA expression. Alterations from miR-29a inhibition to cell culture DNA content by day of proliferation **(A)**. miR-29a inhibition during proliferation or differentiation on the percentage of the total nuclei expressing MYOG **(B)**. miR-29a expression on d 1 of proliferation **(C)** and d 4 of proliferation **(D)**. miR-29a expression from cell cultures treated during proliferation **(E)** or differentiation **(F)**.

Satellite cell culture experiments were conducted to examine loss of function for miR-29a on proliferation and differentiation. Cell proliferation rates were examined daily by measuring DNA content from d 1 to d 4 of the proliferation phase following AntagomiR-29a transfection and compared to Control cultures (Figure 4A). Following transfection on d 1, Lipofectamine treated cultures had reduced (*p* < 0.05) cell numbers when compared to Control. AntagomiR-29a treated cultures did not differ from the control. On d 2 of proliferation AntagomiR-29a 100 nM treated cultures tended (*p* < 0.10) to have reduced DNA content compared to Control cultures. AntagomiR-29a 300 nM treated cultures had increased (*p* < 0.05) DNA content compared to Control cultures on d 3. No cultures differed (*p* > 0.05) from Control cultures on d 4 of proliferation.

AntagomiR-29a treatment effect on satellite cell differentiation ability was assessed with MYOG staining (Figure 4B). Satellite cell differentiation was not altered (*p* > 0.05) by miRNA treatment during proliferation. AntagomiR 29a treatment during differentiation increased (*p* < 0.05) the percentage of nuclei expressing MYOG for AntagomiR 29a 100 and 300 nM treated cells compared to Control cultures. Lipofectamine treated cells did not differ Control cultures.

miR-29a expression was assessed on d 1 and 4 of proliferation and following 4 d of differentiation (Figures 4C–F). AntagomiR-29a at 300 nM reduced (*p* < 0.05) miR-29a expression 24 h following transfection when compared to Control cultures, however AntagomiR-29a 100 nM treated cultures did not differ from Control cells (Figure 4C). Lipofectamine treated cells had increased miR-29a expression on d 1 of proliferation when compared to Control cultures. On d 4 of proliferation AntagomiR-29a 300 nM treated cultures had reduced (*p* < 0.01) miR-29a expression when compared to Control cultures (Figure 4D). Lipofectamine and AntagomiR-29a 100 nM cultures did not differ (*p* > 0.05) from Control cells on d4 of proliferation. Cells treated with either AntagomiR-29a at 100 nM or 300 nM during proliferation had reduced (*p* < 0.01) miR-29a expression following 4 d of differentiation when compared to Control cultures, while Lipofectamine cultures did not differ (*p* > 0.05; Figure 4E). Cells treated with AntagomiR-29a at 100 nM or 300 nM only during the differentiation stage had reduced (*p* < 0.01) miR-29a expression when compared to Control cultures (Figure 4F). Control and Lipofectamine cultures did not differ (*p* > 0.05) from each other on d 4 of differentiation.

## Discussion

Skeletal muscle hypertrophy is an intricate process that spans both the pre and postnatal developmental stages. miRNA are known to be involved in gene regulation and characterizing the transcriptomic changes that occur during development provides a more complete understanding of the mechanisms involved in hypertrophic muscle development. The current study covered a wide developmental range spanning from the conclusion of muscle fiber hyperplasia (PN1; gd 85) to near maturity (MAT). Late gestation (gd 70–140 of gestation) is a period of exponential growth for the fetus and 80% of fetal growth occurs during this period (Rattray et al., 1974). The early postnatal period of development is noted for having increased growth efficiency compared to later postnatal growth (Greenwood and Bell, 2019). Lamb body weight increased as development progressed, with the largest percentage increase (300%) occurring during the transition from prenatal (PN3; gd 133) to postnatal (PW1; d 42) a period of approximately 56 days. Comparatively the post-weaning growth period (PW2; d 65 to MAT; d 243) was the longest at ∼140 d and only resulted in a 97% body weight increase. From PN1 to MAT, muscle weight increased more than total body weight as a percentage, 12,528% and 10,349% respectively, indicating the importance of muscle hypertrophy in overall body growth.

Muscle fiber hypertrophy showed that there was an immense increase in muscle fiber area from PN1 to MAT. During prenatal hypertrophy, PN1 to PN3, Type II fibers were shown to have more hypertrophy from PN1 to PN3 than Type I fibers, 2.7- and 1.9-fold, respectively. Similar results were found during postnatal hypertrophy, PN3 to MAT, with Type II fibers having a 8.9 fold increase and Type I fibers increasing 8.6 fold in size. Within the Type II fibers, Type IIa and IIx showed a similar trend with a 2.6- and 3.0-fold increase during prenatal hypertrophy, and 7.5- and 10.5-fold increase during postnatal hypertrophy, respectively. Type IIax was not found in PN1 (gd 85) samples and could indicate that fiber transition does not start occurring until later in gestation. Postnatal hypertrophy of type IIax fibers was 6.5-fold, the lowest of the all the fibers classified. These findings are consistent with previous studies that show that, while Type I and II fibers are similar in size early in development, Type II fibers surpass Type I fibers in area at maturity (Wegner et al., 2000).

In the current study Type I fiber percentage was greater at PN3 (gd 133) compared to all other developmental time points. In cattle, Type I fiber percentage remained consistent from birth to 24 months of age, however Type IIa fiber percentage decreased with other myosin heavy chain isoforms increasing in percentage (Wegner et al., 2000). Similar findings have been shown in mice and pigs, with all studies noting that the transition from Type I to Type II fiber occurring early in the postnatal period (Wegner et al., 1993; Schiaffino et al., 2015). Muscle fiber type is determined based on the presence of myosin heavy chain isoforms which indicate the metabolic state (oxidative vs. glycolytic) of the individual muscle fibers (Pette and Staron, 1997). In the current study, Type IIa percentage was reduced at maturity compared to prenatal time points, while the percentage of Type IIax fibers increased at maturity. Muscle fiber type is not static and fibers can transition depending on factors like age, nutrition, and activity (Lefaucheur and Gerrard, 2000). Type IIa fibers are more oxidative than Type IIx fibers, so fibers expressing both proteins (Type IIax) would be an intermediate. The dramatic changes in fiber composition in the current study show that while growth is occurring so are metabolic alterations.

miRNA are a group of evolutionarily highly conserved genes that function in post-transcriptional regulation (Horak et al., 2016). The deep sequencing conducted in the current study found 41.80% of the raw reads mapped to known miRNA in the ovine genome. Additionally, a known myomiR, miR-133, was found and was differentially expressed during development. myomiR-1 and -206 were also identified with qPCR and showed increased expression as development progressed. Human and bovine miR-1 and -206 have been identified in ovine samples through sequencing and were shown to be differentially expressed between breeds with higher muscle content compared to local sheep (Kaur et al., 2020). myomiRs are known to be expressed in high abundance in muscle tissue and have significant impact on muscle development (Horak et al., 2016).

miRNA profiles were altered by progressing muscle hypertrophy. There were 184 differentially expressed miRNA, 142 unique miRNA, from all 5 comparisons made (PN1 vs. PN2, PN2 vs. PN3, PN3 vs. PW1, PW1 vs. PW2, PW2 vs. MAT). The prenatal period (PN1 vs. PN2 and PN2 vs. PN3) had more adaptations to the miRNA profile compared to the postnatal period (PW1 vs. PW2 and PW2 vs. MAT; 56 vs. 13), even though the postnatal period was longer (48 vs. 161 d). The transitional period from prenatal to postnatal had the largest number of differentially expressed miRNA (115) reflecting that this period has increased transcriptomic change compared to the other developmental stages examined. Additionally, the transitional stage also had the largest log fold changes indicating that larger magnitude transcriptomic changes were occurring during this period. Similarly, the goat miRNA transcriptome has been characterized from gd 45 to d 90 postnatal at seven time points, with the most differentially expressed miRNA (184) observed from birth to d 90 of age (Ling et al., 2020). Examination of the porcine miRNA transcriptome during fetal development showed that miRNA transcripts increased as fetal muscle development progressed (McDaneld et al., 2009). miR-133 was found in high abundance in adult muscle tissue, and miR-22 and 143 were in abundance in adults and satellite cells. miR-29 was found in muscle and satellite cell cultures, however satellite cell cultures had higher abundance of the miRNA. Postnatally miR-29a was found to be upregulated and miR-127 was down regulated from weaning to maturity in pigs (Chen et al., 2020). A similar trend with miR-29a was found in the current study, however miR-127 was only down regulated during the prenatal to early postnatal comparisons (PN2 vs. PN3, PN3 vs. PW1, and PW1 vs. PW2). miR-22-3p was found in the transitional stage (PN3 vs. PW1) and had the largest log fold change (4.5). miR-22-3p has been previously reported in the skeletal muscle of sheep (Lie et al., 2014; Greene et al., 2019). Overexpression of miR-22-3p promotes myoblast differentiation and reduces proliferation in mouse myoblasts (Marzi et al., 2012). Similar results were seen in porcine satellite cells (Chen et al., 2020). miR-127 plus others have been selected for further testing as important miRNA and their potential role in muscle development and meat quality (Iqbal et al., 2020). miRNA-mRNA networks have shown that expression of miRNA is negatively correlated with the expression of mRNA targets (Ji et al., 2020; Ali et al., 2021).

miR-29a was differentially expressed in 4 of the 5 comparisons made in the current study and continually increased in expression level as development progressed. Ovine satellite cells were cultured to assess the loss of function of miR-29a and its impacts on proliferation and differentiation. miR-29a inhibition reduced cellular proliferation on d 2 but increased proliferation on d 3. AntagomiRs function through binding to the target miRNA and rendering it incapable of binding to mRNA (Broderick and Zamore, 2011; Stenvang et al., 2012). There is a level of cytotoxicity associated with the use of lipofectamine and this could explain initial reductions in cellular proliferation that were seen in all lipofectamine treated cultures (Wang J. Y. et al., 2018). Mouse myoblast, C2C12, proliferation is inhibited by pooled miR-29 family members, however differentiation is promoted (Wei et al., 2013). miR-29 targets Akt3 and reduces the expression of mRNA and protein abundance. Ovine satellite cell differentiation capacity was increased by the inhibition of miR-29a. These results contrast with Wei et al. (2013) and show that even though miRNA family members are similar their biological function can vary between species. miR-29a is highly expressed in beta cells of the pancreatic islets and expression levels are associated with insulin resistance (Dalgaard et al., 2022). miR-29a has been shown to control cell proliferation in small cell lung cancer, hepatocellular carcinoma, and pancreatic ductal adenocarcinoma by targeting a variety of genes depending on cell type (Wang T. et al., 2018). More research is needed to explore the role of miR-29a in skeletal muscle development.

**FIGURE 5:**
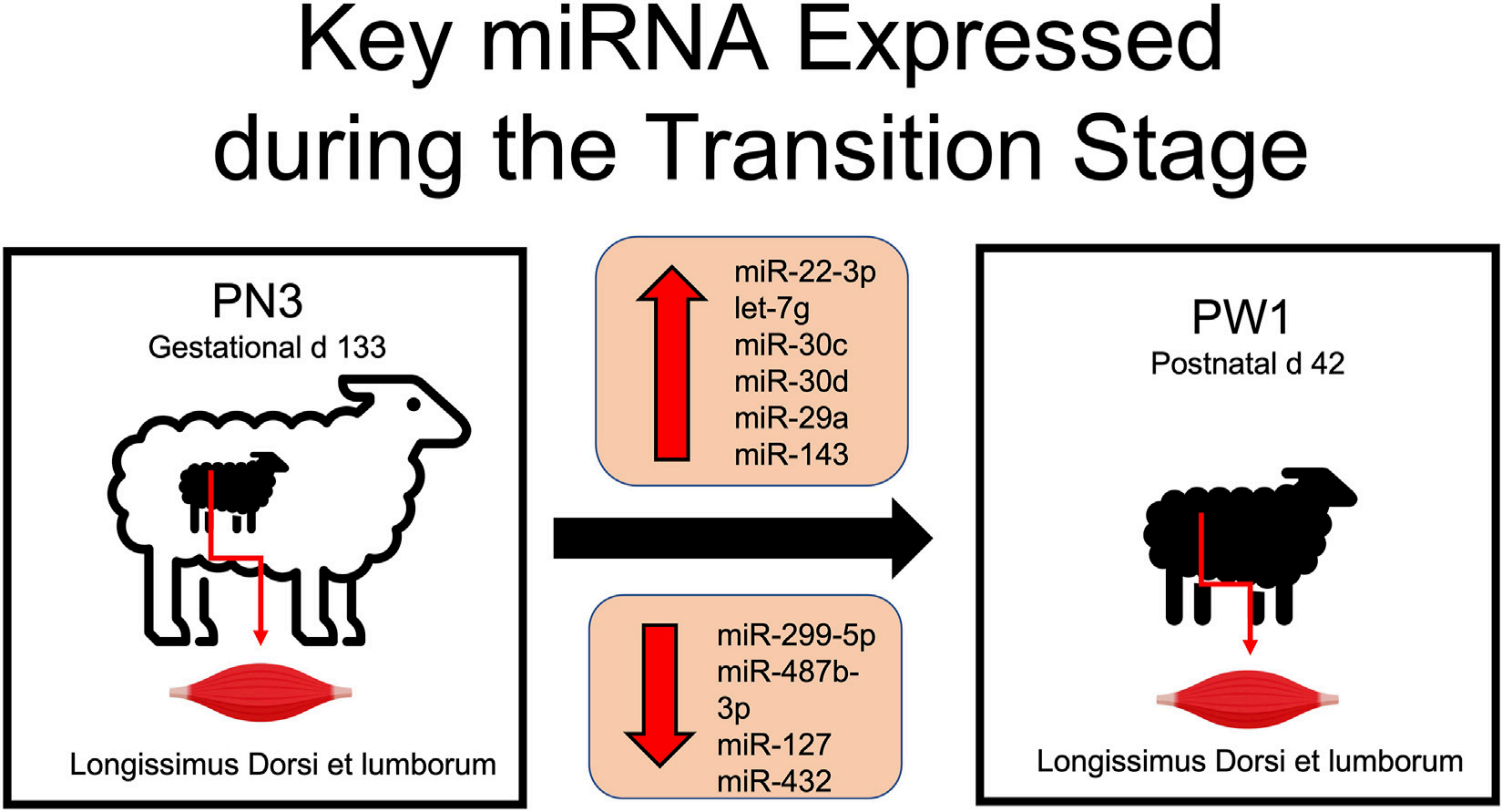
Differentially expressed miRNA involved in skeletal muscle hypertrophy during the transition from prenatal to postnatal development.

Muscle development is essential for both pre and postnatal development of offspring. Muscle fiber characteristics showed massive increases in fiber size and significant changes in muscle fiber type occur during pre and postnatal development. Alterations observed in the miRNA transcriptome add molecular evidence to the magnitude of changes occurring in skeletal muscle tissue during the transitional stage. The greatest change in miRNA expression occurred during the transition from prenatal to postnatal stage with 115 miRNA differentially expressed (DE) in comparison to other developmental time points only having from 2 to 36 DE. Key miRNA identified in this time period included miR-22-3p, -299-5p, -487b-3p, -30c, -127, -432, -30d, -29a, and -143 and let-7g (Figure 5). Of these, several have documented roles in myogenesis (miR-22-3p, -29a, -127, -432, -487b-3p), cardiomyocytes (miR-29a, -30c, -30d or let-7g), vascular smooth muscle (miR-143) or cell proliferation (miR-29a, -299-5p). More research is needed to determine the role of these miRNA in skeletal muscle hypertrophy and if they could be used to alter muscle growth or regeneration.

## Data Availability

The datasets presented in this study can be found in online repositories. The name of the repository and accession number can be found below: NCBI; GSE207055.
