# Supplementary material for: miRNA transcriptome and myofiber characteristics of lamb skeletal muscle during hypertrophic growth1
Source: Front Genet. 2022 Aug 30;13:988756. doi: 10.3389/fgene.2022.988756 (PMC9677349; doi:10.3389/fgene.2022.988756)
Supplement: Supplementary file 1 [file Table1.docx]

**Supplemental Table 1.** The predicted hairpin, mature, and star sequences and position for novel miRNA from sequencing reads.

| Name | Hairpin | Mature | star | **chr** | **Position** | **strand** |
| --- | --- | --- | --- | --- | --- | --- |
| >novel_1 | ugaaaaugacgugacggacuucucgucagccugucuugugagaggcucucacuagucauuuuccc | ugaaaaugacgugacggacuucu | aggcucucacuagucauuuuccc | 18 | 43869442..43869507 | + |
| >novel_10 | guggagcugaucugaguuggagagagaguaguaugacuuccucucucgguuagcuccauag | ccucucucgguuagcuccauag | guggagcugaucugaguuggaga | 26 | 31915780..31915841 | + |
| >novel_100 | gcuuggacucuauggggaaauggagccuugguacuagcagaguccucuugugcccacccaccuccucguagaguucagacga | cuccucguagaguucagacga | gcuuggacucuauggggaaau | 20 | 16629708..16629790 | - |
| >novel_101 | caggggacucuggaggaucagaucaucagugccaauccccugcu | caggggacucuggaggauc | ucagugccaauccccugcu | 11 | 28977365..28977409 | - |
| >novel_102 | cuuccaguuggagagacaagccacagagccacgaggagcuugucucuccaaccaucuugc | cuugucucuccaaccaucuugc | cuuccaguuggagagacaagcc | 3 | 57542374..57542434 | - |
| >novel_104 | cgaggaacucuccuguucgagcguucugcuuguagugugguguggaacguuuaggcaacguggauugcuucuuugcg | aacguggauugcuucuuugcg | cgaggaacucuccugu | 2 | 203485116..203485193 | + |
| >novel_106 | aggggaucuggugccgagacagugucccuuugacucacuucaaggcaaaagaaccccuga | cuucaaggcaaaagaaccccuga | aggggaucuggugccgagacagug | 14 | 1203980..1204040 | + |
| >novel_107 | aaaaaguucauuuggauuuuuccagaagaucuuaagaaauaucugaaugaacuuuuugg | aauaucugaaugaacuuuuugg | aaaaaguucauuuggauuuuuc | 7 | 64136484..64136543 | + |
| >novel_108 | agaaguccauucgggcuuuuccguuacaucuuauggaaacaccgggaugaacuuuuug | aaacaccgggaugaacuuuuug | agaaguccauucgggcuuuucc | 10 | 75655415..75655473 | + |
| >novel_109 | accgcuagaucacaugcagcucagcacaggaacucacugaucugcaugugaucuagugggcu | ucugcaugugaucuagugggcu | accgcuagaucacaugcagcuc | 1 | 139086219..139086281 | + |
| >novel_11 | caguccggucccgcggugucuccgccuguugaugccgcgcggcccgcgggcucggaugcu | caguccggucccgcggugucuccg | gcggcccgcgggcucggaugcu | 1 | 193101027..193101087 | + |
| >novel_110 | agaaaaaugucaugaagaugcuauaacuauaauuucuuauaauaucuucaugacauuuuucuga | uaucuucaugacauuuuucuga | agaaaaaugucaugaagaugcu | 5 | 21678226..21678290 | - |
| >novel_111 | ggaaugagucagugcagaggccggugccacuccaggacagugagccaaggggaggagaaaggcggcagggcacugaaggcug | gcagggcacugaaggcug | ggaaugagucagugcagaggccg | 19 | 16865578..16865660 | + |
| >novel_112 | ugggaagcccugugagaguaacaguaaauacgugcucacuggggcuccagg | auacgugcucacuggggcuccagg | ugggaagcccugugagaguaaca | 10 | 15926098..15926149 | + |
| >novel_113 | ccacacacccccgccgcccccggcacagcccugugacccaggcuggcuuugguggggccugugcggau | ccacacacccccgccgcccc | uuugguggggccugugcggau | 18 | 65228697..65228765 | - |
| >novel_114 | cacugggccggcccuuguuccggcugguacucucaagugcg | ccggcugguacucucaagugcg | cacugggccggcccuug | 26 | 36237381..36237422 | - |
| >novel_115 | aguuucucaugcaggcuuggaaaccuuccuuccaggccuagaugagaaacuuu | ccaggccuagaugagaaacuuu | aguuucucaugcaggcuuggaa | 10 | 25616959..25617012 | + |
| >novel_116 | gcugcacagccgggaggagccgguuucugugcucccg | gccgguuucugugcucccg | gcugcacagccgggag | 5 | 10722829..10722866 | - |
| >novel_117 | gagcuugaggauugcagacuagagaauaaaauagccacaagucucgaucgagcaagcucuc | ucucgaucgagcaagcucuc | gagcuugaggauugcagacu | 2 | 138966943..138967004 | + |
| >novel_118 | gcaaggaagucagggguggaagagagguugguauaucccuuccaucucuacuuucugccu | cuuccaucucuacuuucugccu | gcaaggaagucagggguggaagag | 14 | 51719493..51719553 | - |
| >novel_119 | ucugcagauggcugcauuuccacaguguggaggaagucagucugcagaga | gaggaagucagucugcagaga | ucugcagauggcugcauuucca | 19 | 44129242..44129292 | - |
| >novel_12 | aaaaguucauuuggguuguuccuuaacaguuuaugggaaaaccugaaugaacuuauugg | aaaaccugaaugaacuuauugg | aaaaguucauuuggguuguuccu | 12 | 49020364..49020423 | - |
| >novel_120 | cagugaaccagcugacagcucaaauccuauagucuuuggcuguacaacugguucauugac | uggcuguacaacugguucauugac | cagugaaccagcugacagcucaaa | 5 | 58428346..58428406 | + |
| >novel_121 | ucucgccuuggccugcaguggaggggacgcgaaagugauuuaaccucugcagaacccugggcagaca | ucugcagaacccugggcagaca | ucucgccuuggccugcagugg | 12 | 73585149..73585216 | + |
| >novel_122 | gagagcccggauagcucagucgguagagcaucagacuuuuaaucugaggguccaggguucaaguc | gagagcccggauagcucagucggu | ucugaggguccaggguucaaguc | 11 | 27322407..27322472 | + |
| >novel_123 | agaacccccggccagaaguuugcagcugucuaacaucugggcgagaguucucc | aacaucugggcgagaguucucc | agaacccccggccagaaguuug | 1 | 105045744..105045797 | - |
| >novel_124 | ccggcccuggccgccacccgcggcugaugcugccgcugcggaggggcggggggccggggccggga | cggggggccggggccggga | ccggcccuggccgccaccc | 8 | 12603683..12603748 | - |
| >novel_126 | ccuuccacagguacagucccugugaguccagcagagugccugcugcucugugugggaacuggaccugcaggagggac | aacuggaccugcaggagggac | ccuuccacagguacaguccc | 17 | 67911218..67911295 | + |
| >novel_127 | agacauugaguacucccuguuuaauaggucaggagaccuagggcuguggggaccucucaugucugu | uguggggaccucucaugucugu | agacauugaguacucccuguuua | 19 | 52476619..52476685 | + |
| >novel_128 | aaagaauuugcuuggguuuucauaaaauguuauggaaaaaucugagugaacuuuuugg | aaaaucugagugaacuuuuugg | aaagaauuugcuuggguuuuca | 15 | 24080069..24080127 | - |
| >novel_129 | aaaagagcuguugaugaacuaguuaucuccaaacaguuuauccacagcuuuucauc | aaaagagcuguugaugaacuagu | caguuuauccacagcuuuucauc | 3 | 130874904..130874960 | - |
| >novel_13 | uaugccucagaaugaucuauuuguauuguaaaaaaaucuaaauagaucauucugaagcauagu | auagaucauucugaagcauagu | uaugccucagaaugaucuauu | 1 | 105931867..105931930 | + |
| >novel_130 | uagauacuaggauagagacaaagugaaugggucucuguccuaguaucucg | uagauacuaggauagagaca | ggucucuguccuaguaucucg | 14 | 34141650..34141700 | - |
| >novel_131 | acagaguucugggcccugcucagaacuacugagucagaaucucugagagcagggcccagaaaucuguau | acagaguucugggcccugcuca | agcagggcccagaaaucuguau | 10 | 15312083..15312152 | + |
| >novel_132 | cuuccguacuguuugagccaccagggaagcccuuuuggaggcuuuccagguagcgcuagug | cuuccguacuguuugagc | uuuccagguagcgcuagug | 6 | 98696622..98696683 | - |
| >novel_133 | agucugcugucucaggggugucguuuggcucuagguccccuugagacaguagaaaca | uccccuugagacaguagaaaca | agucugcugucucaggggugucg | 11 | 40109309..40109366 | + |
| >novel_134 | uuacccguccuuccuuccucacagauugcuguccagagauuuacuucucuugacagccuguugagggccucagucuggauguccaagg | uuacccguccuuccuuccucac | gagggccucagucuggauguccaagg | 24 | 26227407..26227495 | - |
| >novel_135 | ucucuauagcuacuuagaccaaggaacuuacuacugucuaaguagucguggccgagugg | aguagucguggccgagugg | ucucuauagcuacuua | 20 | 30114379..30114438 | - |
| >novel_136 | aaaaaguucauuuggguuucugcauaugcuauuacagcaaaaccugaacgaacuuuuugu | caaaaccugaacgaacuuuuugu | aaaaaguucauuuggguuucugca | 3 | 145451659..145451719 | - |
| >novel_137 | auugccgaaagccuuuccccguguuuggaggguccauccugucccuugcaaacccuggugcuuucguacaugu | cuggugcuuucguacaugu | auugccgaaagccuuuccccgug | 13 | 66932653..66932726 | - |
| >novel_138 | aggggggguggaaggggaggcguggaaaucagcuucuugucuggacuggucc | cuucuugucuggacuggucc | aggggggguggaaggggaggcg | 24 | 18664570..18664622 | - |
| >novel_139 | aaugauauagacucuagacuugugcuacaucaguuaguacagaucuagaguuuauaucauuua | aaugauauagacucuagacuug | gaucuagaguuuauaucauuua | 7 | 33196520..33196583 | - |
| >novel_14 | cauuccaaggcuugccauucaucuggcuaaacuugugauuggcacgucuuggaauga | auuggcacgucuuggaauga | cauuccaaggcuugccauuca | X | 83608712..83608769 | - |
| >novel_140 | uaggucauuucaaagagggcugaugggaccaaauccuggagcccucaacgcugugaccaaag | uaggucauuucaaagagggcugaug | ggagcccucaacgcugugaccaaag | 21 | 579984..580046 | + |
| >novel_141 | gccuagacagguacuaaucauguagaccauguguaaucaguaucugucuggguag | aaucaguaucugucuggguag | gccuagacagguacuaaucau | X | 84475313..84475368 | + |
| >novel_142 | aggugguuucucgaaaucgaaacuagacugagcuuucugaagcgcuuccugggaccaaccuga | cuuccugggaccaaccuga | aggugguuucucgaaauc | 11 | 52408804..52408867 | + |
| >novel_143 | ggcgggggcggcggguggacggacggcucagguugccccucugacgccugcucuggcccc | ucugacgccugcucuggcccc | ggcgggggcggcggguggacg | 6 | 114787213..114787273 | - |
| >novel_144 | acuaaaaauuccacaugcucuauaacaaagugaaucagcuguacagcauguagaaucuuugguug | cagcauguagaaucuuugguug | acuaaaaauuccacaugcucua | 3 | 204766102..204766167 | + |
| >novel_145 | uuugagggcaagacugaguuuauucuguuuuuggagaacaagcucaguguccucugaag | uuugagggcaagacugaguuuauuc | acaagcucaguguccucugaag | 26 | 14387454..14387513 | - |
| >novel_146 | ucagcaagaugguuggagagacaagcuccucguggcucuguggcuugucucuccaacuggaaggcagaga | ucagcaagaugguuggagagaca | ucucuccaacuggaaggcagaga | 3 | 57542372..57542442 | + |
| >novel_147 | aaaaguuuguuuugaguuuucccaaaagauaguauggaagaaccugaacgaaccuuuug | aagaaccugaacgaaccuuuug | aaaaguuuguuuugaguuuuccc | X | 82384535..82384594 | - |
| >novel_149 | aguucuguagucaaagugacuucuuccaugaggaauagaaauugcuuuggcuucagaaucca | aguucuguagucaaagugacuu | auugcuuuggcuucagaaucca | 20 | 29946032..29946094 | - |
| >novel_15 | uagucucccguugucacuggucuuaagagggcagaagaccagcuggacuggggacuagg | uagucucccguugucacuggucu | accagcuggacuggggacuagg | 7 | 33068802..33068861 | + |
| >novel_150 | uuggcagguguaguuuaaagcgugugcuuaagccucuauuuuccugaaaaguaccgcuuucugccuuaccagu | uuggcagguguaguuuaaagcgugu | uaccgcuuucugccuuaccagu | 11 | 42696543..42696616 | + |
| >novel_151 | aggaagaagaagcagaguuagacauagcuaacauguuugucuguccucucuucucuuccaga | ucuguccucucuucucuuccaga | aggaagaagaagcagaguuagaca | 26 | 22595763..22595825 | + |
| >novel_153 | uuugcucugggcugaagaccacuccaacagcccucauuguaugggagccagcccagggcaacca | uuugcucugggcugaagacc | gagccagcccagggcaacca | 15 | 29362104..29362168 | - |
| >novel_154 | ggaucuaguuccccagugcaggggaccuggguuccccgcauuggagaacuagauccca | ccgcauuggagaacuagauccca | ggaucuaguuccccagugcaggg | X | 19188744..19188802 | - |
| >novel_156 | uagaaaguuucuuugggguuuuucaguaagaucagauagaaaaccucaaacgaacuuuuuggc | uagaaaguuucuuugggguuuu | aaccucaaacgaacuuuuuggc | 2 | 70661764..70661827 | - |
| >novel_157 | accgccuuuguguugcccauucacuuuuggaaacuaguggauguggugucaaaaaaggcguaca | accgccuuuguguugcccauucacu | uggauguggugucaaaaaaggcguaca | 7 | 1534289..1534353 | - |
| >novel_158 | uccuccuacuccucugcuaucuuguuaaggagucaagacagcugagaaguaggacaaug | acagcugagaaguaggacaaug | uccuccuacuccucugcuaucu | 18 | 1265196..1265255 | - |
| >novel_159 | uuugcuaacauaccucaaaaaacaauuuugaaaaaguacauaugacuuuuugcaccuuuuaaaaauugagguguaguuagcaaaca | aauugagguguaguuagcaaaca | uuugcuaacauaccucaaaaaa | 12 | 1577914..1578000 | + |
| >novel_16 | ccaggagugucuuuggauugucuggagccugggcaauucugagaacuccugcaa | ccaggagugucuuuggauuguc | caauucugagaacuccugcaa | 19 | 11797291..11797345 | + |
| >novel_161 | agugucuaguuuggggccgggaugcggagcucuugacugagcggguaggccaagcggaggcuucagggacaucgg | agugucuaguuuggggccgggau | gccaagcggaggcuucagggacaucgg | 17 | 44945688..44945763 | - |
| >novel_162 | ugaccucccgccucuggacagggucuugccuugggcucuggucggggggcaggagguggug | ugaccucccgccucuggacaggg | cuggucggggggcaggagguggug | 12 | 40793737..40793798 | - |
| >novel_163 | ugccuagagagguucuguuuacuucaacuacugguaaacagagccuuccuggaucga | ugccuagagagguucuguuuacu | uaaacagagccuuccuggaucga | X | 84477860..84477917 | + |
| >novel_165 | gauucagagacagaaaagcuguguccugguccauaacccuuugcuuuucugcucuugagaauccc | gauucagagacagaaaagcugugu | uuugcuuuucugcucuugagaauccc | 13 | 67317518..67317583 | + |
| >novel_166 | ccugucacugaccacuguucuagugguuaggauucgac | uagugguuaggauucgac | ccugucacugaccacuguu | 15 | 16347824..16347862 | + |
| >novel_168 | cucggccugcggugccuccgcucagcgagucugugcucgcacucaggcgcccgggcuccgucgagcg | cucggccugcggugccuccgc | ucaggcgcccgggcuccgucgagcg | 18 | 65957072..65957139 | + |
| >novel_169 | gagacagguuucugccuugcuucgucuuuauguauuuccaagaaacaagacugaaaccugccuuuc | aacaagacugaaaccugccuuuc | gagacagguuucugccuugcuuc | 1 | 4212564..4212630 | + |
| >novel_17 | aaugugggagagagugccgccuccagcgucacucacucgccaccc | aaugugggagagagugccgccu | agcgucacucacucgccaccc | 11 | 36011033..36011078 | + |
| >novel_170 | agacaucccugauggugaaaugguuaagauucugcccuuucacuguagaggaugcaagu | uuucacuguagaggaugcaagu | agacaucccugauggugaaaug | 9 | 3034514..3034573 | + |
| >novel_171 | ccggacccugguuagaaugccaagaaagccacagcugguuuuuuuuuuuuuggcuauucuguaggggcgccu | ccggacccugguuagaaugcca | gcuauucuguaggggcgccu | 3 | 220415706..220415778 | - |
| >novel_172 | aaaaaauuugcuuggauuuuucuguaagauauuaugggaaaaccagaacgaacuuuuuga | aaaaccagaacgaacuuuuuga | aaaaaauuugcuuggauuuuuc | 21 | 10217930..10217990 | + |
| >novel_173 | caaagaguucaugcagguuuuugaaaaaucugaacgaacucuuuggc | aaaaucugaacgaacucuuuggc | caaagaguucaugcagguuuuug | 3 | 42718633..42718680 | + |
| >novel_174 | cucucuguucagaagucaauaguugaguauugaagaagucuguuauugacuauugacuucugaacagagagca | auugacuucugaacagagagca | cucucuguucagaagucaauag | 12 | 12449946..12450019 | + |
| >novel_175 | aagcuacuggaaccuguucugcuucugggcaggauccuauuucucugcccccuuuucagcucagcuuuuccccaaaauuugc | aagcuacuggaaccuguucu | cucagcuuuuccccaaaauuugc | 2 | 35182005..35182087 | - |
| >novel_176 | aaugagauccaaagcuggaugaugguuucucauccaacuuugaaucucaacuu | auccaacuuugaaucucaacuu | aaugagauccaaagcuggauga | 6 | 25717773..25717826 | + |
| >novel_177 | uccgauuuucuucaccuucuguuuuguuuucuuccccaguuuuuagaaaucugaaggaaaaaaauuc | uccgauuuucuucaccuucuguuu | uuagaaaucugaaggaaaaaaauuc | 12 | 1533569..1533636 | - |
| >novel_178 | cacugcaguguccacucuguucuaggauggacucgcugcagaagc | ggauggacucgcugcagaagc | cacugcaguguccacucugu | 25 | 30750454..30750499 | - |
| >novel_179 | gcccagccaugugccccagugcucaccucucugggcuugguggcacccagacugugcuggagcug | cagacugugcuggagcug | gcccagccaugugccc | 2 | 236139005..236139070 | + |
| >novel_18 | ucugagagacaugaucuuguuuuguucaacucagacuccaaaacaagaucacgccucucaga | aaacaagaucacgccucucaga | ucugagagacaugaucuuguuu | 21 | 12354371..12354433 | + |
| >novel_180 | cugcucuuguugacucuuaagaggcugaaaaguuucaucuuuucguucgccuggaagggcauaguaaggguggca | cugcucuuguugacucuuaag | gaagggcauaguaaggguggca | 2 | 81206774..81206849 | - |
| >novel_181 | gacacugccaggcccaaggcuguuccccuugcucugaggcagugucgu | uugcucugaggcagugucgu | gacacugccaggcccaagg | 18 | 64628337..64628385 | + |
| >novel_182 | ggagggccaggcuucuggaaucacaguuauuuggaauuuggggauucugauaggcgugguucucauu | auucugauaggcgugguucucauu | ggagggccaggcuucuggaauca | 2 | 24883159..24883226 | - |
| >novel_19 | uagagaagcgcugggggaaagccuuggacgugaggugcugccccuugaugcuugucuuca | ugccccuugaugcuugucuuca | uagagaagcgcugggggaaagc | 22 | 32550933..32550993 | + |
| >novel_20 | uuuuguuccagauuuccagcuguauuuguacaaauucggcugcaaacaggaacaaga | uuuuguuccagauuuccagcugua | ucggcugcaaacaggaacaaga | 3 | 214311026..214311083 | + |
| >novel_21 | augccuuccucuaaacagucagcagaauuuugcugacuguuuagaggaagagaua | cugacuguuuagaggaagagaua | augccuuccucuaaacagucagc | 20 | 38041246..38041301 | - |
| >novel_22 | caggauaaguugagagagcguucuuccgcucccuucgauagcucagcuggu | gcucccuucgauagcucagcuggu | caggauaaguugagagagcgu | 9 | 43292770..43292821 | + |
| >novel_23 | aagagguucauucaggguuuucuguaacaucuaauggaaaacccugaacgaaggucuugg | aaacccugaacgaaggucuugg | aagagguucauucaggguuuuc | 12 | 76518253..76518313 | - |
| >novel_24 | ugcuuagacugguacugaccauguagaccauguguaaucaguaucugucuggguaga | aaucaguaucugucuggguaga | ugcuuagacugguacugacc | X | 84549939..84549996 | + |
| >novel_25 | uccuccuccuccgcccaacuugggugaagcgcccagcuggguggggagguggcgcu | uccuccuccuccgcccaacuug | cagcuggguggggagguggcgcu | 12 | 78332291..78332347 | - |
| >novel_26 | ugcaggucuuaucagaaggagcaaguggaacuuuagcucuuucuguaggacuuguccuu | ugcaggucuuaucagaaggagc | ucuuucuguaggacuuguccuu | 26 | 24006236..24006295 | - |
| >novel_27 | agucccugggcugcaggcgcugcugcuacgcauacacacaacggcugccgcucagugggc | agucccugggcugcaggcgcug | aacggcugccgcucagugggc | 7 | 41743465..41743525 | + |
| >novel_28 | cuucuuccgugacuguuggacggacauuccuuugggaaaagcuuucuccacagugagguacgaagacgg | cuucuuccgugacuguugga | acagugagguacgaagacgg | 15 | 41431351..41431420 | + |
| >novel_29 | cacgacugaagugacuucgauacugcugcugcgaagucacuucagucguauc | cacgacugaagugacuucgaua | gcgaagucacuucagucguauc | 5 | 58254070..58254122 | - |
| >novel_3 | aaguacaggaugcccaaugaauuaaauccaucuaaauacauuucacugggcauccucugcuuu | ucacugggcauccucugcuuu | aaguacaggaugcccaaugaau | 18 | 64106173..64106236 | - |
| >novel_30* | augguccaacacucgccuaaaggaaaucucacauaguaauuuccuuuaggcgaguguuggaccauac | uuuaggcgaguguuggaccauac | augguccaacacucgccuaaagg | 2 | 121675399..121675466 | + |
| >novel_31 | ugaaaaguuuguucggguuuuuccauaagcuguuauggaaagauguuuuuccauaacagcuuauggaaaaacccaaugaacuuuuuggcc | ugaaaaguuuguucggguuuuu | aaacccaaugaacuuuuuggcc | 16 | 22502398..22502488 | - |
| >novel_32 | cucggucuagaccucuaagucuagaaccccucugauucaggauucucugagucaggauucuaggcuuagaggucauagaccaagag | cucggucuagaccucuaagucu | gcuuagaggucauagaccaagag | 3 | 162941902..162941988 | - |
| >novel_33 | ugugccauucgggaucuuuaguuggguccaaaugcuaaagaucccgaauggcacgag | ugugccauucgggaucuuuagu | uaaagaucccgaauggcacgag | 13 | 60375244..60375301 | - |
| >novel_35 | uugucccuggucgcggccucugcaaggucaaaugucaccgcggggccgcgggcaggggucaggu | uugucccuggucgcggccucugca | cggggccgcgggcaggggucaggu | 5 | 5592893..5592957 | + |
| >novel_36 | cugcucuuucucucccagcgucugggucacaggcauguggcagugucggcccggccgcccgggcugaaggagcgug | gccgcccgggcugaaggagcgug | cugcucuuucucucccagcgucug | 12 | 42854186..42854262 | + |
| >novel_37 | accugggcccggccgggcaaggcggugagucucuccucgccggcccaccaggacccaggcgg | cggcccaccaggacccaggcgg | accugggcccggccgggcaaggc | 14 | 52400687..52400749 | - |
| >novel_38 | ugcgaagaagaggccugacguguguguuggggcagauagguuccaggcucacucaggcccccucuuccagc | ugcgaagaagaggccugacgugugu | ucacucaggcccccucuuccagc | 25 | 31006695..31006766 | + |
| >novel_39 | uuggacaagacugagcgaguugaagugggguucaguucaguucacucgcucagugguguccgaau | uuggacaagacugagcgaguug | acucgcucagugguguccgaau | 5 | 41279156..41279221 | + |
| >novel_4 | cuagaguuugacggccucggcccucccuguuccagguggaggccugcaaacuguaggg | uggaggccugcaaacuguaggg | cuagaguuugacggccucggcccu | 11 | 61834932..61834990 | + |
| >novel_40 | aagacagagggagagcugguuugcuugcuugccugccagaacauccaggucuacgucugucuucu | uccaggucuacgucugucuucu | aagacagagggagagcugguuu | 20 | 39501542..39501607 | + |
| >novel_41 | cauguccggucugcagagcuagacuucugguccggacacuggacccccuggucugcaggccuggaccuggg | cauguccggucugcagagcuaga | uggucugcaggccuggaccuggg | 8 | 87962814..87962885 | - |
| >novel_42 | uuaauaucuggcauagaauguuuaugucuauaauaaauguucuguaccagauauuaacu | uuaauaucuggcauagaauguu | uguucuguaccagauauuaacu | 10 | 32579676..32579735 | + |
| >novel_44 | aaaaguucguucugguuuuccugugcacaguauggaagaacccgagugaacuuuugg | aagaacccgagugaacuuuugg | aaaaguucguucugguuuuccu | 8 | 82411812..82411869 | - |
| >novel_45 | ugacuuagcacacacauauauguauauauguguauguaaauauauauauguguguacuaaggagcu | uauauguguguacuaaggagcu | ugacuuagcacacacauauaug | 2 | 225276793..225276859 | + |
| >novel_47 | agucuaugagucaggauacuuuaagucauuugugaauuaaacagcauauaacuuaaaguauccugacucauagacuu | aaguauccugacucauagacuu | agucuaugagucaggauacuuu | 1 | 165454056..165454133 | - |
| >novel_48 | gaaaguguaaggcaaggucugguuccuuccugcuucaccagccuucccucaaacuuucuu | gaaaguguaaggcaaggucuggu | cagccuucccucaaacuuucuu | 15 | 23791900..23791960 | - |
| >novel_49 | uacagacaacggauauguagacauucguugaaggaauauccguugucuguaaaguaga | auccguugucuguaaaguaga | uacagacaacggauaugu | 6 | 6659248..6659306 | - |
| >novel_5 | ucccgggcuggaggagucugcagggaggggacugcgucucgucccgcccggggag | ucccgggcuggaggagucugca | cgucucgucccgcccggggag | 14 | 60548859..60548914 | + |
| >novel_50 | cccggggagugaguggagccguggguguugucggcggggccgccuggcuccgcugagucccgcucuugccuccagcuggguc | cccggggagugaguggagccg | gcucuugccuccagcuggguc | 19 | 12417763..12417845 | - |
| >novel_52 | cccacgcccgggccagccccaggcggggcguggggauggaggga | cccacgcccgggccagcc | cggggcguggggauggaggga | 1 | 21772493..21772537 | - |
| >novel_54 | uggcaucagggugcagccugggagcuuaguucaccccacggcugcagacugggucaga | cacggcugcagacugggucaga | uggcaucagggugcagccuggg | JH922574.1 | 4237..4295 | + |
| >novel_55 | uggguuuccgacugccucuccgcuuccucgggggaaagcggggaggggucggggaccccua | uggguuuccgacugccucuccgc | ggggaggggucggggaccccua | 1 | 127747741..127747802 | + |
| >novel_56 | cagggugagaauuuugcugggcaucuucuacaacuucccagccaaauccccacccucug | cagggugagaauuuugcug | gccaaauccccacccucug | 18 | 47219420..47219479 | - |
| >novel_57 | aaaaguucauucagguuuuuccguaacguauuguggacaaaucugaaugaauuuucug | acaaaucugaaugaauuuucug | aaaaguucauucagguuuuucc | 3 | 215712472..215712530 | + |
| >novel_58 | cagauggauuaggccaaagugucgcuuauuuaugcauauacacuuugaccuguaacccaucugga | cuuugaccuguaacccaucugga | cagauggauuaggccaaagug | 11 | 52582654..52582719 | + |
| >novel_59 | aaaagguucauuuggauuuuuccacagcaucuuacggagaaaucugaaugaaccuuuugg | gaaaucugaaugaaccuuuugg | aaaagguucauuuggauuuuuc | 8 | 28284805..28284865 | + |
| >novel_6 | uacccauccaggugcaguucauguagaaucaauguacugugccacggauggguagc | uacugugccacggauggguagc | uacccauccaggugcaguuca | X | 84543634..84543690 | + |
| >novel_60 | cuccccuccgcuugcuccuccagagaagcgucgcuuuccgagcaagauggauuggacgggcggggccgggggug | cgggcggggccgggggug | cuccccuccgcuugcu | 11 | 36131428..36131502 | + |
| >novel_61 | aaaaaguucauuugggcuuuuucguaagauguuacagaaaaaccuugaugaacuuuuuga | aaaaccuugaugaacuuuuuga | aaaaaguucauuugggcuuuuuc | 4 | 85934151..85934211 | - |
| >novel_62 | uaaggggcugagaccuacucuguucuccacgagcgccagaaggaagcagccccuuggu | uaaggggcugagaccuacucuguu | cgccagaaggaagcagccccuuggu | 17 | 64993505..64993563 | + |
| >novel_63 | agaaaguucauuugguuuuccuguaagcugagaaaaccggcaugaacuuuuugg | aaaccggcaugaacuuuuugg | agaaaguucauuugguuuuc | 18 | 59088360..59088414 | - |
| >novel_64 | aggggacugaggugggcgcagugagaaaggagccauggccuuuaggcgccguguguccgucucuuccccaga | guguguccgucucuuccccaga | aggggacugaggugggcgcagug | 15 | 29407229..29407301 | - |
| >novel_65 | ucgugggacagaguggagggaggauccaugcucaccaggcccuccgucccuucuugcccaccagu | uccgucccuucuugcccaccagu | ucgugggacagaguggagggagg | 3 | 216496203..216496268 | + |
| >novel_66 | gagggagaaggauggaguuggguggccacagcucuuagccucagcaccauccuucccuggca | ucagcaccauccuucccuggca | gagggagaaggauggaguugggu | 14 | 124558686..24558748 | + |
| >novel_67 | ugggugcccagugaagucaguguuccucauuggcauuacugagcaucuagu | uuggcauuacugagcaucuagu | ugggugcccagugaagucagug | 25 | 5456294..5456345 | - |
| >novel_68 | cuggucgggcugcugcaggcucagaucggcagggcagucaggguggag | cuggucgggcugcugcaggcuc | gcagggcagucaggguggag | 14 | 667840..667888 | + |
| >novel_69 | ccaaaaaguucguucugauauuuccguaauaucuuaugggaaaaugcaaacgaacuuuuuggcc | augcaaacgaacuuuuuggcc | ccaaaaaguucguucugauau | 3 | 184953672..184953736 | - |
| >novel_7 | acccugacgggcguggauuguggggaaauuaacugacaacacgcgucagucaggcucuc | acccugacgggcguggauugug | acacgcgucagucaggcucuc | 15 | 52475108..52475167 | + |
| >novel_70 | auggggagacguccacgguuagggugcacccucugacugucuggugucuucccag | ugacugucuggugucuucccag | auggggagacguccacgguuag | 12 | 78291711..78291766 | - |
| >novel_71 | acugaacgaccagggaauucccuccaaauucuuuuuauucugggaauucccuggcaguucagucc | acugaacgaccagggaauuccc | gaauucccuggcaguucagucc | 13 | 33768236..33768301 | - |
| >novel_72 | uccagacggugcuggcucucugaguauuuauucgccaucauccuggagccacgccuucuuggaga | uccagacggugcuggcucucu | ggagccacgccuucuuggaga | 21 | 42474383..42474448 | + |
| >novel_73 | caaccagguccacugauuugcgaucaugcuuauaucucaaaucaguggaccugguag | cucaaaucaguggaccugguag | caaccagguccacugauuugcg | 3 | 69488166..69488223 | + |
| >novel_74 | aauggggcugugcuauaacccaggagcucggaaaauuaaaaucugggucagugcccagcuccaucuuc | aauggggcugugcuauaacc | ucagugcccagcuccaucuuc | 23 | 22309904..22309972 | - |
| >novel_75 | caucuuacugggcagcauuggauggugucuggucucuaauacugccugguaaugaugac | uaauacugccugguaaugaugac | caucuuacugggcagcauugga | 12 | 49397879..49397938 | - |
| >novel_76 | cuccagguugaagggcugcgcuuucccucguuaucgagggcggccggacuuuggggaacucgc | cuccagguugaagggcug | gccggacuuuggggaacucgc | 6 | 85955657..85955720 | - |
| >novel_77 | guuacaaagagucagacaugacuuagugacuggugguggugguuuaguugcuaagucgugucugacucaaaccc | cgugucugacucaaaccc | guuacaaagagucagacaugac | 6 | 13606029..13606103 | + |
| >novel_78 | uggaugaguucaaaaguuuuucuccccaaucugagaaaaacuuuugaacucauccaaa | aaaacuuuugaacucauccaaa | uggaugaguucaaaaguuuuuc | 12 | 18667633..18667691 | + |
| >novel_79 | uucggcgccaccacccugcgggucgcgcuguaagaucuugccccgcggguguucgcccaccu | uucggcgccaccacccugcggg | cgcggguguucgcccaccu | 25 | 8590653..8590715 | - |
| >novel_8 | aaaaguucauuuggguuguuccuuaacagcuuaugggaaaaccugaaugaacuuaucgg | aaaaccugaaugaacuuaucgg | aaaaguucauuuggguuguuccu | X | 120613659..120613718 | - |
| >novel_80 | uugacuacuggaagaaccagguauguauagucaaagcuauggguuuuccaguagucaugu | uugacuacuggaagaaccaggu | uauggguuuuccaguagucaugu | 16 | 40292223..40292283 | + |
| >novel_81 | aaguucauuuggguguuuccguaagguguuagggaaaagccugaaugaacucuuug | aaaagccugaaugaacucuuug | aaguucauuuggguguuuccgu | 23 | 22826201..22826257 | - |
| >novel_82 | uggaagacgaacuuagagaugcuguugcaaaggcaguaaugguuuagacagcaccagugcuuccgaacagcaucucuaaguucgucuuccgua | uggaagacgaacuuagagaugcu | caucucuaaguucgucuuccgua | 19 | 40409712..40409805 | - |
| >novel_83 | uugcacaacucuagaagacauguuccauauuguaaaucgugucucucagaguugugcaaaa | uugcacaacucuagaagacaug | ugucucucagaguugugcaaaa | 24 | 31351267..31351328 | + |
| >novel_84 | cgucgcccgcgucccccccccggggccgcggccgcggcgcgccc | cggggccgcggccgcggcgcgccc | cgucgcccgcgucccccc | 15 | 80862581..80862625 | + |
| >novel_85 | agcagcuugccugaggucaguggcucugagccgaccuuagagcaagcugccca | cgaccuuagagcaagcugccca | agcagcuugccugaggucaguggc | 21 | 44918404..44918457 | - |
| >novel_86 | uacccagacaggugcaguucacguagaauugguauacugugccacagcuggguaga | uacugugccacagcuggguaga | uacccagacaggugcaguuca | X | 84548418..84548474 | + |
| >novel_87 | cuagaucugguguggugcugcccuugccuggggcucaacgcccuggcaggcgccacauaaauggauggauag | cuagaucugguguggugcugcccu | gcaggcgccacauaaauggauggauag | 12 | 61574961..61575033 | - |
| >novel_88 | agagucggacacgacugagcgacuucacuaugugcuuagucacucagucgugcccgaauuacu | ucacucagucgugcccgaauuacu | agagucggacacgacugagcgacu | 3 | 161873548..161873611 | + |
| >novel_89 | ucaggaguuggggaugggguggcguggucugugaccucucccugucuucaauccuguagu | ucccugucuucaauccuguagu | ucaggaguuggggauggggugg | 14 | 981235..981295 | - |
| >novel_90 | aauguucauucgagcuuuuccuuaagcuguuauggagaaacuagaaugaacuuuuu | agaaacuagaaugaacuuuuu | aauguucauucgagcuuuucc | 21 | 10119788..10119844 | + |
| >novel_91 | cugucucugcuguuaggcuucggcaggcgcucgaucaggaggccugcggccuggccuuggagaagccaugga | cugucucugcuguuaggcuu | gccuggccuuggagaagccaugga | 2 | 142467164..142467236 | + |
| >novel_92 | ugaggcgagggcccagaggacacugcagagaugggaauaaccacucuuguucuccuggucucuucccucagu | uucuccuggucucuucccucagu | ugaggcgagggcccagaggaca | 24 | 26275759..26275831 | - |
| >novel_93 | ugucuguucagaauaucuaucagaauaucuuuaauagauauucugaacagacaac | ugucuguucagaauaucuau | auagauauucugaacagacaac | 9 | 81662712..81662767 | + |
| >novel_94 | uggucuuuggcgguuaauccauauuguagcauguggauuaaccucaaaagaccaua | uggucuuuggcgguuaaucca | gauuaaccucaaaagaccaua | 3 | 33004542..33004598 | - |
| >novel_96 | acugcggccugucuuagggacccucagguaaccagacugucccugugacagugagcggc | ccugugacagugagcggc | acugcggccugucuuagggac | 9 | 2770960..2771019 | + |
| >novel_97 | gcuuuuggagagagaagacggcgguuugccgacaauucucggccaaagggccuuuuguuuuugcggagguaa | gcuuuuggagagagaagacgg | cuuuuguuuuugcggagguaa | 5 | 37217911..37217983 | - |
| >novel_99 | aucuggaucagucuaugcugacuuugugauucagggucagcaugaccugguccccacau | aucuggaucagucuaugcugacu | ucagcaugaccugguccccacau | 11 | 7267484..7267543 | - |

**Supplemental Table 2.** The enriched Gene Ontology terms for mRNA targets of differentially expressed miRNA from PN1 (gestational d 85) v PN2 (gestational d 110) comparison.

| **GO_accession** | **Description** | **Term_type** | **Corr. *P-*Value** |
| --- | --- | --- | --- |
| GO:0003824 | catalytic activity | molecular_function | 7.99E-13 |
| GO:0008152 | metabolic process | biological_process | 7.99E-13 |
| GO:0005488 | binding | molecular_function | 6.10E-10 |
| GO:0016787 | hydrolase activity | molecular_function | 6.07E-09 |
| GO:0043167 | ion binding | molecular_function | 1.02E-08 |
| GO:0005975 | carbohydrate metabolic process | biological_process | 2.26E-08 |
| GO:0071704 | organic substance metabolic process | biological_process | 3.17E-08 |
| GO:0044238 | primary metabolic process | biological_process | 3.52E-08 |
| GO:0008827 | cytochrome o ubiquinol oxidase activity | molecular_function | 9.89E-08 |
| GO:0016682 | oxidoreductase activity, acting on diphenols and related substances as donors, oxygen as acceptor | molecular_function | 1.22E-07 |
| GO:0005623 | cell | cellular_component | 8.77E-07 |
| GO:0044464 | cell part | cellular_component | 8.77E-07 |
| GO:0043227 | membrane-bounded organelle | cellular_component | 9.33E-07 |
| GO:0043231 | intracellular membrane-bounded organelle | cellular_component | 1.13E-06 |
| GO:0044710 | single-organism metabolic process | biological_process | 1.30E-06 |
| GO:0005575 | cellular_component | cellular_component | 3.58E-06 |
| GO:0044237 | cellular metabolic process | biological_process | 5.07E-06 |
| GO:0005622 | intracellular | cellular_component | 7.39E-06 |
| GO:0055114 | oxidation-reduction process | biological_process | 7.46E-06 |
| GO:0070011 | peptidase activity, acting on L-amino acid peptides | molecular_function | 7.70E-06 |
| GO:0008233 | peptidase activity | molecular_function | 9.78E-06 |
| GO:0044424 | intracellular part | cellular_component | 1.04E-05 |
| GO:0004175 | endopeptidase activity | molecular_function | 1.06E-05 |
| GO:0097159 | organic cyclic compound binding | molecular_function | 1.12E-05 |
| GO:1901363 | heterocyclic compound binding | molecular_function | 1.12E-05 |
| GO:0016679 | oxidoreductase activity, acting on diphenols and related substances as donors | molecular_function | 1.95E-05 |
| GO:0043168 | anion binding | molecular_function | 2.97E-05 |
| GO:0036094 | small molecule binding | molecular_function | 4.04E-05 |
| GO:0015002 | heme-copper terminal oxidase activity | molecular_function | 0.00011357 |
| GO:0043170 | macromolecule metabolic process | biological_process | 0.00011357 |
| GO:0000166 | nucleotide binding | molecular_function | 0.00011545 |
| GO:1901265 | nucleoside phosphate binding | molecular_function | 0.00011545 |
| GO:0043234 | protein complex | cellular_component | 0.00020054 |
| GO:0032559 | adenyl ribonucleotide binding | molecular_function | 0.00042369 |
| GO:0046483 | heterocycle metabolic process | biological_process | 0.00042802 |
| GO:0030554 | adenyl nucleotide binding | molecular_function | 0.00048968 |
| GO:0004252 | serine-type endopeptidase activity | molecular_function | 0.00048968 |
| GO:0005524 | ATP binding | molecular_function | 0.00048968 |
| GO:0006807 | nitrogen compound metabolic process | biological_process | 0.00053022 |
| GO:0051179 | localization | biological_process | 0.00053022 |
| GO:0016491 | oxidoreductase activity | molecular_function | 0.00055176 |
| GO:1901360 | organic cyclic compound metabolic process | biological_process | 0.00058033 |
| GO:0043169 | cation binding | molecular_function | 0.00058235 |
| GO:0032553 | ribonucleotide binding | molecular_function | 0.00069947 |
| GO:0006725 | cellular aromatic compound metabolic process | biological_process | 0.00073281 |
| GO:0032555 | purine ribonucleotide binding | molecular_function | 0.00073281 |
| GO:0046872 | metal ion binding | molecular_function | 0.00073281 |
| GO:0017076 | purine nucleotide binding | molecular_function | 0.00073281 |
| GO:0001883 | purine nucleoside binding | molecular_function | 0.00073281 |
| GO:0032549 | ribonucleoside binding | molecular_function | 0.00073281 |
| GO:0032550 | purine ribonucleoside binding | molecular_function | 0.00073281 |
| GO:0034641 | cellular nitrogen compound metabolic process | biological_process | 0.00081892 |
| GO:0035639 | purine ribonucleoside triphosphate binding | molecular_function | 0.000848 |
| GO:0001882 | nucleoside binding | molecular_function | 0.00095933 |
| GO:0090304 | nucleic acid metabolic process | biological_process | 0.0010025 |
| GO:0006139 | nucleobase-containing compound metabolic process | biological_process | 0.00103 |
| GO:0044723 | single-organism carbohydrate metabolic process | biological_process | 0.0015994 |
| GO:0005634 | nucleus | cellular_component | 0.0016026 |
| GO:0044260 | cellular macromolecule metabolic process | biological_process | 0.0016247 |
| GO:0043226 | organelle | cellular_component | 0.0016622 |
| GO:0051641 | cellular localization | biological_process | 0.001719 |
| GO:0006265 | DNA topological change | biological_process | 0.001719 |
| GO:0051234 | establishment of localization | biological_process | 0.001719 |
| GO:0043229 | intracellular organelle | cellular_component | 0.0018905 |
| GO:0006810 | transport | biological_process | 0.0019743 |
| GO:0008150 | biological_process | biological_process | 0.0025402 |
| GO:0006259 | DNA metabolic process | biological_process | 0.0037645 |
| GO:0051649 | establishment of localization in cell | biological_process | 0.0040851 |
| GO:0016820 | hydrolase activity, acting on acid anhydrides, catalyzing transmembrane movement of substances | molecular_function | 0.0040851 |
| GO:0008238 | exopeptidase activity | molecular_function | 0.0041978 |
| GO:0008236 | serine-type peptidase activity | molecular_function | 0.0053134 |
| GO:0017171 | serine hydrolase activity | molecular_function | 0.0053134 |
| GO:0006091 | generation of precursor metabolites and energy | biological_process | 0.0053959 |
| GO:0022900 | electron transport chain | biological_process | 0.0056467 |
| GO:0016817 | hydrolase activity, acting on acid anhydrides | molecular_function | 0.0077224 |
| GO:0000271 | polysaccharide biosynthetic process | biological_process | 0.0077224 |
| GO:0046914 | transition metal ion binding | molecular_function | 0.0078126 |
| GO:0005976 | polysaccharide metabolic process | biological_process | 0.010408 |
| GO:0019222 | regulation of metabolic process | biological_process | 0.012333 |
| GO:0033036 | macromolecule localization | biological_process | 0.012733 |
| GO:0042623 | ATPase activity, coupled | molecular_function | 0.013153 |
| GO:0005576 | extracellular region | cellular_component | 0.014085 |
| GO:0016798 | hydrolase activity, acting on glycosyl bonds | molecular_function | 0.014657 |
| GO:0000228 | nuclear chromosome | cellular_component | 0.015792 |
| GO:0031323 | regulation of cellular metabolic process | biological_process | 0.016234 |
| GO:0051704 | multi-organism process | biological_process | 0.016528 |
| GO:0008094 | DNA-dependent ATPase activity | molecular_function | 0.017519 |
| GO:0004553 | hydrolase activity, hydrolyzing O-glycosyl compounds | molecular_function | 0.017942 |
| GO:0044421 | extracellular region part | cellular_component | 0.021641 |
| GO:0003916 | DNA topoisomerase activity | molecular_function | 0.021951 |
| GO:0003723 | RNA binding | molecular_function | 0.022197 |
| GO:0008104 | protein localization | biological_process | 0.023475 |
| GO:0044262 | cellular carbohydrate metabolic process | biological_process | 0.027172 |
| GO:0017111 | nucleoside-triphosphatase activity | molecular_function | 0.027172 |
| GO:0009405 | pathogenesis | biological_process | 0.029164 |
| GO:0080090 | regulation of primary metabolic process | biological_process | 0.029656 |
| GO:0016462 | pyrophosphatase activity | molecular_function | 0.029817 |
| GO:0016757 | transferase activity, transferring glycosyl groups | molecular_function | 0.030267 |
| GO:0046906 | tetrapyrrole binding | molecular_function | 0.030779 |
| GO:0044264 | cellular polysaccharide metabolic process | biological_process | 0.032623 |
| GO:0005515 | protein binding | molecular_function | 0.03278 |
| GO:0044422 | organelle part | cellular_component | 0.03278 |
| GO:0006950 | response to stress | biological_process | 0.033105 |
| GO:0006508 | proteolysis | biological_process | 0.033105 |
| GO:0048519 | negative regulation of biological process | biological_process | 0.035424 |
| GO:0046907 | intracellular transport | biological_process | 0.035516 |
| GO:0044446 | intracellular organelle part | cellular_component | 0.036574 |
| GO:0019219 | regulation of nucleobase-containing compound metabolic process | biological_process | 0.039444 |
| GO:0051171 | regulation of nitrogen compound metabolic process | biological_process | 0.039444 |
| GO:0020037 | heme binding | molecular_function | 0.042301 |
| GO:0018995 | host | cellular_component | 0.042301 |
| GO:0043245 | extraorganismal space | cellular_component | 0.042301 |
| GO:0043657 | host cell | cellular_component | 0.042301 |
| GO:0044215 | other organism | cellular_component | 0.042301 |
| GO:0044216 | other organism cell | cellular_component | 0.042301 |
| GO:0044217 | other organism part | cellular_component | 0.042301 |
| GO:0016818 | hydrolase activity, acting on acid anhydrides, in phosphorus-containing anhydrides | molecular_function | 0.042301 |
| GO:0005737 | cytoplasm | cellular_component | 0.049643 |
| GO:0033692 | cellular polysaccharide biosynthetic process | biological_process | 0.049898 |

**Supplemental Table 3.** The enriched Gene Ontology terms for mRNA targets of differentially expressed miRNA from PN2 (gestational d 110) v PN3 (gestational d 133) comparison.

| **GO_accession** | **Description** | **Term_type** | **Corr. *P-*Value** |
| --- | --- | --- | --- |
| GO:0003824 | catalytic activity | molecular_function | 1.21E-16 |
| GO:0008152 | metabolic process | biological_process | 4.69E-14 |
| GO:0016787 | hydrolase activity | molecular_function | 1.73E-11 |
| GO:0005488 | binding | molecular_function | 1.54E-09 |
| GO:0070011 | peptidase activity, acting on L-amino acid peptides | molecular_function | 2.80E-09 |
| GO:0008233 | peptidase activity | molecular_function | 2.80E-09 |
| GO:0071704 | organic substance metabolic process | biological_process | 9.16E-09 |
| GO:0044238 | primary metabolic process | biological_process | 1.03E-08 |
| GO:0004175 | endopeptidase activity | molecular_function | 4.99E-08 |
| GO:0043227 | membrane-bounded organelle | cellular_component | 7.03E-08 |
| GO:0043231 | intracellular membrane-bounded organelle | cellular_component | 1.17E-07 |
| GO:0005975 | carbohydrate metabolic process | biological_process | 1.30E-07 |
| GO:0043167 | ion binding | molecular_function | 2.58E-07 |
| GO:0008827 | cytochrome o ubiquinol oxidase activity | molecular_function | 1.83E-06 |
| GO:0005623 | cell | cellular_component | 2.03E-06 |
| GO:0044464 | cell part | cellular_component | 2.03E-06 |
| GO:0044710 | single-organism metabolic process | biological_process | 2.09E-06 |
| GO:0016682 | oxidoreductase activity, acting on diphenols and related substances as donors, oxygen as acceptor | molecular_function | 2.18E-06 |
| GO:0005622 | intracellular | cellular_component | 4.07E-06 |
| GO:0005575 | cellular_component | cellular_component | 5.17E-06 |
| GO:0044237 | cellular metabolic process | biological_process | 6.02E-06 |
| GO:0055114 | oxidation-reduction process | biological_process | 6.16E-06 |
| GO:0043234 | protein complex | cellular_component | 1.30E-05 |
| GO:0044424 | intracellular part | cellular_component | 1.57E-05 |
| GO:0016491 | oxidoreductase activity | molecular_function | 1.65E-05 |
| GO:0043170 | macromolecule metabolic process | biological_process | 1.84E-05 |
| GO:0097159 | organic cyclic compound binding | molecular_function | 2.27E-05 |
| GO:1901363 | heterocyclic compound binding | molecular_function | 2.27E-05 |
| GO:1901360 | organic cyclic compound metabolic process | biological_process | 3.10E-05 |
| GO:0046483 | heterocycle metabolic process | biological_process | 3.34E-05 |
| GO:0006508 | proteolysis | biological_process | 5.15E-05 |
| GO:0006725 | cellular aromatic compound metabolic process | biological_process | 0.00011966 |
| GO:0015002 | heme-copper terminal oxidase activity | molecular_function | 0.00016289 |
| GO:0006807 | nitrogen compound metabolic process | biological_process | 0.00018018 |
| GO:0034641 | cellular nitrogen compound metabolic process | biological_process | 0.00020128 |
| GO:0051179 | localization | biological_process | 0.00025706 |
| GO:0006265 | DNA topological change | biological_process | 0.00026937 |
| GO:0006139 | nucleobase-containing compound metabolic process | biological_process | 0.00039627 |
| GO:0016679 | oxidoreductase activity, acting on diphenols and related substances as donors | molecular_function | 0.00039824 |
| GO:0016798 | hydrolase activity, acting on glycosyl bonds | molecular_function | 0.00047039 |
| GO:0004553 | hydrolase activity, hydrolyzing O-glycosyl compounds | molecular_function | 0.00066942 |
| GO:0051234 | establishment of localization | biological_process | 0.00084738 |
| GO:0090304 | nucleic acid metabolic process | biological_process | 0.0009167 |
| GO:0006810 | transport | biological_process | 0.00093589 |
| GO:0043226 | organelle | cellular_component | 0.0011104 |
| GO:0044422 | organelle part | cellular_component | 0.0011186 |
| GO:0005576 | extracellular region | cellular_component | 0.0012125 |
| GO:0043229 | intracellular organelle | cellular_component | 0.0015703 |
| GO:0044446 | intracellular organelle part | cellular_component | 0.0015703 |
| GO:0008236 | serine-type peptidase activity | molecular_function | 0.0016074 |
| GO:0017171 | serine hydrolase activity | molecular_function | 0.0016074 |
| GO:0004252 | serine-type endopeptidase activity | molecular_function | 0.0016355 |
| GO:0005634 | nucleus | cellular_component | 0.0017288 |
| GO:0046872 | metal ion binding | molecular_function | 0.0029354 |
| GO:0043169 | cation binding | molecular_function | 0.0029492 |
| GO:0008238 | exopeptidase activity | molecular_function | 0.0039118 |
| GO:0003723 | RNA binding | molecular_function | 0.0041173 |
| GO:0036094 | small molecule binding | molecular_function | 0.0041521 |
| GO:0044421 | extracellular region part | cellular_component | 0.0042033 |
| GO:0033036 | macromolecule localization | biological_process | 0.0042033 |
| GO:0003916 | DNA topoisomerase activity | molecular_function | 0.0049689 |
| GO:0000166 | nucleotide binding | molecular_function | 0.0052822 |
| GO:1901265 | nucleoside phosphate binding | molecular_function | 0.0052822 |
| GO:0043168 | anion binding | molecular_function | 0.0052822 |
| GO:0046914 | transition metal ion binding | molecular_function | 0.0054727 |
| GO:0032559 | adenyl ribonucleotide binding | molecular_function | 0.0064789 |
| GO:0044723 | single-organism carbohydrate metabolic process | biological_process | 0.0068465 |
| GO:0005524 | ATP binding | molecular_function | 0.0069445 |
| GO:0051641 | cellular localization | biological_process | 0.0069445 |
| GO:0051649 | establishment of localization in cell | biological_process | 0.007389 |
| GO:0022900 | electron transport chain | biological_process | 0.007389 |
| GO:0044260 | cellular macromolecule metabolic process | biological_process | 0.0082796 |
| GO:0006091 | generation of precursor metabolites and energy | biological_process | 0.0086476 |
| GO:0005044 | scavenger receptor activity | molecular_function | 0.0092776 |
| GO:0038024 | cargo receptor activity | molecular_function | 0.0092776 |
| GO:0016757 | transferase activity, transferring glycosyl groups | molecular_function | 0.0093267 |
| GO:0030554 | adenyl nucleotide binding | molecular_function | 0.0093279 |
| GO:0004571 | mannosyl-oligosaccharide 1,2-alpha-mannosidase activity | molecular_function | 0.0093279 |
| GO:0015924 | mannosyl-oligosaccharide mannosidase activity | molecular_function | 0.0093279 |
| GO:0033643 | host cell part | cellular_component | 0.0094064 |
| GO:0018995 | host | cellular_component | 0.010544 |
| GO:0043245 | extraorganismal space | cellular_component | 0.010544 |
| GO:0043657 | host cell | cellular_component | 0.010544 |
| GO:0044215 | other organism | cellular_component | 0.010544 |
| GO:0044216 | other organism cell | cellular_component | 0.010544 |
| GO:0044217 | other organism part | cellular_component | 0.010544 |
| GO:0008150 | biological_process | biological_process | 0.015985 |
| GO:0071702 | organic substance transport | biological_process | 0.016158 |
| GO:0008270 | zinc ion binding | molecular_function | 0.017853 |
| GO:0003676 | nucleic acid binding | molecular_function | 0.019939 |
| GO:0000228 | nuclear chromosome | cellular_component | 0.019983 |
| GO:0006259 | DNA metabolic process | biological_process | 0.021134 |
| GO:0008104 | protein localization | biological_process | 0.021825 |
| GO:0012505 | endomembrane system | cellular_component | 0.021825 |
| GO:0051704 | multi-organism process | biological_process | 0.022015 |
| GO:0019222 | regulation of metabolic process | biological_process | 0.023644 |
| GO:0000271 | polysaccharide biosynthetic process | biological_process | 0.026647 |
| GO:0032555 | purine ribonucleotide binding | molecular_function | 0.026647 |
| GO:0032553 | ribonucleotide binding | molecular_function | 0.026647 |
| GO:0042025 | host cell nucleus | cellular_component | 0.026647 |
| GO:0001883 | purine nucleoside binding | molecular_function | 0.026647 |
| GO:0032549 | ribonucleoside binding | molecular_function | 0.026647 |
| GO:0032550 | purine ribonucleoside binding | molecular_function | 0.026647 |
| GO:0046907 | intracellular transport | biological_process | 0.026759 |
| GO:0008094 | DNA-dependent ATPase activity | molecular_function | 0.027738 |
| GO:0004197 | cysteine-type endopeptidase activity | molecular_function | 0.028199 |
| GO:0035639 | purine ribonucleoside triphosphate binding | molecular_function | 0.028199 |
| GO:0019538 | protein metabolic process | biological_process | 0.028469 |
| GO:0015031 | protein transport | biological_process | 0.029487 |
| GO:0001882 | nucleoside binding | molecular_function | 0.032191 |
| GO:0045184 | establishment of protein localization | biological_process | 0.032622 |
| GO:0051701 | interaction with host | biological_process | 0.034748 |
| GO:0005515 | protein binding | molecular_function | 0.034748 |
| GO:0017076 | purine nucleotide binding | molecular_function | 0.034824 |
| GO:0031323 | regulation of cellular metabolic process | biological_process | 0.03709 |
| GO:0016070 | RNA metabolic process | biological_process | 0.039522 |
| GO:0005976 | polysaccharide metabolic process | biological_process | 0.040799 |
| GO:0080090 | regulation of primary metabolic process | biological_process | 0.044601 |
| GO:0030234 | enzyme regulator activity | molecular_function | 0.045762 |
| GO:0015923 | mannosidase activity | molecular_function | 0.047447 |
| GO:0009405 | pathogenesis | biological_process | 0.049978 |

**Supplemental Table 4.** The enriched Gene Ontology terms for mRNA targets of differentially expressed miRNA from PN3 (gestational d 133) v PW1 (d 42) comparison.

| **GO_accession** | **Description** | **Term_type** | **Corr. *P-*Value** |
| --- | --- | --- | --- |
| GO:0003824 | catalytic activity | molecular_function | 2.07E-20 |
| GO:0008152 | metabolic process | biological_process | 1.36E-14 |
| GO:0016787 | hydrolase activity | molecular_function | 4.09E-12 |
| GO:0005488 | binding | molecular_function | 1.89E-10 |
| GO:0008233 | peptidase activity | molecular_function | 2.51E-09 |
| GO:0070011 | peptidase activity, acting on L-amino acid peptides | molecular_function | 5.17E-09 |
| GO:0071704 | organic substance metabolic process | biological_process | 5.19E-09 |
| GO:0044238 | primary metabolic process | biological_process | 6.72E-09 |
| GO:0005975 | carbohydrate metabolic process | biological_process | 1.27E-08 |
| GO:0043167 | ion binding | molecular_function | 2.81E-08 |
| GO:0004175 | endopeptidase activity | molecular_function | 5.13E-08 |
| GO:0008827 | cytochrome o ubiquinol oxidase activity | molecular_function | 1.30E-07 |
| GO:0043227 | membrane-bounded organelle | cellular_component | 2.27E-07 |
| GO:0016682 | oxidoreductase activity, acting on diphenols and related substances as donors, oxygen as acceptor | molecular_function | 2.68E-07 |
| GO:0043231 | intracellular membrane-bounded organelle | cellular_component | 3.35E-07 |
| GO:0043234 | protein complex | cellular_component | 1.02E-06 |
| GO:0043168 | anion binding | molecular_function | 2.14E-06 |
| GO:0044237 | cellular metabolic process | biological_process | 2.87E-06 |
| GO:0036094 | small molecule binding | molecular_function | 2.87E-06 |
| GO:0000166 | nucleotide binding | molecular_function | 3.77E-06 |
| GO:1901265 | nucleoside phosphate binding | molecular_function | 3.77E-06 |
| GO:0097159 | organic cyclic compound binding | molecular_function | 3.77E-06 |
| GO:1901363 | heterocyclic compound binding | molecular_function | 3.77E-06 |
| GO:0005623 | cell | cellular_component | 4.56E-06 |
| GO:0044464 | cell part | cellular_component | 4.56E-06 |
| GO:0051179 | localization | biological_process | 5.43E-06 |
| GO:0032559 | adenyl ribonucleotide binding | molecular_function | 8.18E-06 |
| GO:0005524 | ATP binding | molecular_function | 8.62E-06 |
| GO:0043170 | macromolecule metabolic process | biological_process | 1.27E-05 |
| GO:0051234 | establishment of localization | biological_process | 1.27E-05 |
| GO:0030554 | adenyl nucleotide binding | molecular_function | 1.27E-05 |
| GO:0006810 | transport | biological_process | 1.38E-05 |
| GO:0005622 | intracellular | cellular_component | 1.38E-05 |
| GO:0032555 | purine ribonucleotide binding | molecular_function | 2.11E-05 |
| GO:0055114 | oxidation-reduction process | biological_process | 2.28E-05 |
| GO:0032553 | ribonucleotide binding | molecular_function | 2.43E-05 |
| GO:0001883 | purine nucleoside binding | molecular_function | 2.43E-05 |
| GO:0032549 | ribonucleoside binding | molecular_function | 2.43E-05 |
| GO:0032550 | purine ribonucleoside binding | molecular_function | 2.43E-05 |
| GO:0035639 | purine ribonucleoside triphosphate binding | molecular_function | 2.57E-05 |
| GO:0017076 | purine nucleotide binding | molecular_function | 2.69E-05 |
| GO:0016679 | oxidoreductase activity, acting on diphenols and related substances as donors | molecular_function | 5.67E-05 |
| GO:0001882 | nucleoside binding | molecular_function | 5.67E-05 |
| GO:0051641 | cellular localization | biological_process | 6.27E-05 |
| GO:0044710 | single-organism metabolic process | biological_process | 6.28E-05 |
| GO:0015002 | heme-copper terminal oxidase activity | molecular_function | 7.23E-05 |
| GO:0006265 | DNA topological change | biological_process | 0.00010799 |
| GO:0033036 | macromolecule localization | biological_process | 0.00013844 |
| GO:0046483 | heterocycle metabolic process | biological_process | 0.00013844 |
| GO:1901360 | organic cyclic compound metabolic process | biological_process | 0.00017519 |
| GO:0044424 | intracellular part | cellular_component | 0.00018385 |
| GO:0051649 | establishment of localization in cell | biological_process | 0.00023694 |
| GO:0005575 | cellular_component | cellular_component | 0.00024188 |
| GO:0034641 | cellular nitrogen compound metabolic process | biological_process | 0.00028553 |
| GO:0006725 | cellular aromatic compound metabolic process | biological_process | 0.00039521 |
| GO:0016491 | oxidoreductase activity | molecular_function | 0.00042559 |
| GO:0006807 | nitrogen compound metabolic process | biological_process | 0.00042559 |
| GO:0044422 | organelle part | cellular_component | 0.00095452 |
| GO:0004252 | serine-type endopeptidase activity | molecular_function | 0.00097036 |
| GO:0006508 | proteolysis | biological_process | 0.0010008 |
| GO:0044723 | single-organism carbohydrate metabolic process | biological_process | 0.0010265 |
| GO:0000271 | polysaccharide biosynthetic process | biological_process | 0.0011366 |
| GO:0044446 | intracellular organelle part | cellular_component | 0.0011963 |
| GO:0006139 | nucleobase-containing compound metabolic process | biological_process | 0.0012078 |
| GO:0006091 | generation of precursor metabolites and energy | biological_process | 0.0012078 |
| GO:0090304 | nucleic acid metabolic process | biological_process | 0.0013768 |
| GO:0008236 | serine-type peptidase activity | molecular_function | 0.0014771 |
| GO:0017171 | serine hydrolase activity | molecular_function | 0.0014771 |
| GO:0022900 | electron transport chain | biological_process | 0.0017947 |
| GO:0044421 | extracellular region part | cellular_component | 0.0022202 |
| GO:0005515 | protein binding | molecular_function | 0.0024158 |
| GO:0071702 | organic substance transport | biological_process | 0.0024798 |
| GO:0008104 | protein localization | biological_process | 0.0025429 |
| GO:0004553 | hydrolase activity, hydrolyzing O-glycosyl compounds | molecular_function | 0.0026576 |
| GO:0008238 | exopeptidase activity | molecular_function | 0.0027409 |
| GO:0016798 | hydrolase activity, acting on glycosyl bonds | molecular_function | 0.0027409 |
| GO:0019222 | regulation of metabolic process | biological_process | 0.0027525 |
| GO:0016757 | transferase activity, transferring glycosyl groups | molecular_function | 0.0033881 |
| GO:0016817 | hydrolase activity, acting on acid anhydrides | molecular_function | 0.0036186 |
| GO:0044260 | cellular macromolecule metabolic process | biological_process | 0.0039838 |
| GO:0005044 | scavenger receptor activity | molecular_function | 0.0047623 |
| GO:0038024 | cargo receptor activity | molecular_function | 0.0047623 |
| GO:0046907 | intracellular transport | biological_process | 0.0047755 |
| GO:0015031 | protein transport | biological_process | 0.0048727 |
| GO:0005576 | extracellular region | cellular_component | 0.00515 |
| GO:0045184 | establishment of protein localization | biological_process | 0.0055396 |
| GO:0031323 | regulation of cellular metabolic process | biological_process | 0.0062713 |
| GO:0000502 | proteasome complex | cellular_component | 0.0062713 |
| GO:0005976 | polysaccharide metabolic process | biological_process | 0.0062713 |
| GO:0043226 | organelle | cellular_component | 0.0065664 |
| GO:0070008 | serine-type exopeptidase activity | molecular_function | 0.0078861 |
| GO:0003916 | DNA topoisomerase activity | molecular_function | 0.0078861 |
| GO:0043229 | intracellular organelle | cellular_component | 0.0087566 |
| GO:0005634 | nucleus | cellular_component | 0.0087566 |
| GO:0016820 | hydrolase activity, acting on acid anhydrides, catalyzing transmembrane movement of substances | molecular_function | 0.013005 |
| GO:0080090 | regulation of primary metabolic process | biological_process | 0.014026 |
| GO:0016740 | transferase activity | molecular_function | 0.01476 |
| GO:0031090 | organelle membrane | cellular_component | 0.014929 |
| GO:0043169 | cation binding | molecular_function | 0.015907 |
| GO:0033692 | cellular polysaccharide biosynthetic process | biological_process | 0.015907 |
| GO:0004571 | mannosyl-oligosaccharide 1,2-alpha-mannosidase activity | molecular_function | 0.018891 |
| GO:0015924 | mannosyl-oligosaccharide mannosidase activity | molecular_function | 0.018891 |
| GO:0034613 | cellular protein localization | biological_process | 0.018937 |
| GO:0070727 | cellular macromolecule localization | biological_process | 0.018937 |
| GO:0046872 | metal ion binding | molecular_function | 0.020375 |
| GO:0044264 | cellular polysaccharide metabolic process | biological_process | 0.021161 |
| GO:0051704 | multi-organism process | biological_process | 0.02172 |
| GO:0017111 | nucleoside-triphosphatase activity | molecular_function | 0.024225 |
| GO:0019219 | regulation of nucleobase-containing compound metabolic process | biological_process | 0.024225 |
| GO:0051171 | regulation of nitrogen compound metabolic process | biological_process | 0.024225 |
| GO:0008150 | biological_process | biological_process | 0.024225 |
| GO:0018995 | host | cellular_component | 0.024225 |
| GO:0043245 | extraorganismal space | cellular_component | 0.024225 |
| GO:0043657 | host cell | cellular_component | 0.024225 |
| GO:0044215 | other organism | cellular_component | 0.024225 |
| GO:0044216 | other organism cell | cellular_component | 0.024225 |
| GO:0044217 | other organism part | cellular_component | 0.024225 |
| GO:0012505 | endomembrane system | cellular_component | 0.024955 |
| GO:0016070 | RNA metabolic process | biological_process | 0.026179 |
| GO:0044765 | single-organism transport | biological_process | 0.026651 |
| GO:0003723 | RNA binding | molecular_function | 0.026651 |
| GO:0016462 | pyrophosphatase activity | molecular_function | 0.028176 |
| GO:0006886 | intracellular protein transport | biological_process | 0.029287 |
| GO:0009405 | pathogenesis | biological_process | 0.033 |
| GO:0016836 | hydro-lyase activity | molecular_function | 0.033982 |
| GO:0046914 | transition metal ion binding | molecular_function | 0.035407 |
| GO:0060255 | regulation of macromolecule metabolic process | biological_process | 0.035407 |
| GO:0032991 | macromolecular complex | cellular_component | 0.035721 |
| GO:0051262 | protein tetramerization | biological_process | 0.038268 |
| GO:0008092 | cytoskeletal protein binding | molecular_function | 0.040143 |
| GO:0033643 | host cell part | cellular_component | 0.040143 |
| GO:0015923 | mannosidase activity | molecular_function | 0.040399 |
| GO:0004127 | cytidylate kinase activity | molecular_function | 0.042501 |
| GO:0000228 | nuclear chromosome | cellular_component | 0.046094 |
| GO:0016818 | hydrolase activity, acting on acid anhydrides, in phosphorus-containing anhydrides | molecular_function | 0.047134 |
| GO:0010468 | regulation of gene expression | biological_process | 0.048499 |
| GO:0016192 | vesicle-mediated transport | biological_process | 0.048538 |
| GO:0008270 | zinc ion binding | molecular_function | 0.048538 |

**Supplemental Table 5.** The enriched Gene Ontology terms for mRNA targets of differentially expressed miRNA from PW1 (d 42) v PW2 (d 65) comparison.

| **GO_accession** | **Description** | **Term_type** | **Corr. *P-*Value** |
| --- | --- | --- | --- |
| GO:0016787 | hydrolase activity | molecular_function | 0.0045419 |
| GO:0008152 | metabolic process | biological_process | 0.0045419 |
| GO:0003824 | catalytic activity | molecular_function | 0.0085778 |
| GO:0005975 | carbohydrate metabolic process | biological_process | 0.01039 |

**Supplemental Table 6.** The enriched Gene Ontology terms for mRNA targets of differentially expressed miRNA from PW2 (d 65) v MAT (d 203) comparison.

| **GO_accession** | **Description** | **Term_type** | **Corr. *P-*Value** |
| --- | --- | --- | --- |
| GO:0003824 | catalytic activity | molecular_function | 2.28E-12 |
| GO:0008152 | metabolic process | biological_process | 1.03E-11 |
| GO:0071704 | organic substance metabolic process | biological_process | 2.95E-09 |
| GO:0044238 | primary metabolic process | biological_process | 1.25E-08 |
| GO:0044237 | cellular metabolic process | biological_process | 2.30E-07 |
| GO:0016787 | hydrolase activity | molecular_function | 3.05E-06 |
| GO:0005575 | cellular_component | cellular_component | 4.27E-06 |
| GO:0005975 | carbohydrate metabolic process | biological_process | 5.82E-06 |
| GO:0005623 | cell | cellular_component | 6.17E-06 |
| GO:0044464 | cell part | cellular_component | 6.17E-06 |
| GO:0043227 | membrane-bounded organelle | cellular_component | 7.13E-06 |
| GO:0043167 | ion binding | molecular_function | 7.13E-06 |
| GO:0043231 | intracellular membrane-bounded organelle | cellular_component | 8.33E-06 |
| GO:0044424 | intracellular part | cellular_component | 8.77E-06 |
| GO:0070011 | peptidase activity, acting on L-amino acid peptides | molecular_function | 9.82E-06 |
| GO:0005622 | intracellular | cellular_component | 9.82E-06 |
| GO:0008233 | peptidase activity | molecular_function | 1.07E-05 |
| GO:0044710 | single-organism metabolic process | biological_process | 1.21E-05 |
| GO:0004175 | endopeptidase activity | molecular_function | 1.51E-05 |
| GO:0043234 | protein complex | cellular_component | 2.02E-05 |
| GO:0016682 | oxidoreductase activity, acting on diphenols and related substances as donors, oxygen as acceptor | molecular_function | 2.23E-05 |
| GO:0043170 | macromolecule metabolic process | biological_process | 2.23E-05 |
| GO:0005488 | binding | molecular_function | 2.23E-05 |
| GO:0008827 | cytochrome o ubiquinol oxidase activity | molecular_function | 2.63E-05 |
| GO:0051179 | localization | biological_process | 3.12E-05 |
| GO:0033036 | macromolecule localization | biological_process | 0.00015429 |
| GO:0005576 | extracellular region | cellular_component | 0.00015429 |
| GO:1901360 | organic cyclic compound metabolic process | biological_process | 0.00015429 |
| GO:0046483 | heterocycle metabolic process | biological_process | 0.00017706 |
| GO:0034641 | cellular nitrogen compound metabolic process | biological_process | 0.00025228 |
| GO:0006807 | nitrogen compound metabolic process | biological_process | 0.00025228 |
| GO:0015002 | heme-copper terminal oxidase activity | molecular_function | 0.00046473 |
| GO:0097159 | organic cyclic compound binding | molecular_function | 0.00046473 |
| GO:1901363 | heterocyclic compound binding | molecular_function | 0.00046473 |
| GO:0006725 | cellular aromatic compound metabolic process | biological_process | 0.00058377 |
| GO:0051234 | establishment of localization | biological_process | 0.0005866 |
| GO:0044260 | cellular macromolecule metabolic process | biological_process | 0.00065026 |
| GO:0055114 | oxidation-reduction process | biological_process | 0.00065026 |
| GO:0006810 | transport | biological_process | 0.00067617 |
| GO:0043226 | organelle | cellular_component | 0.00070081 |
| GO:0043229 | intracellular organelle | cellular_component | 0.00089108 |
| GO:0071702 | organic substance transport | biological_process | 0.0010011 |
| GO:0033643 | host cell part | cellular_component | 0.0010011 |
| GO:0016679 | oxidoreductase activity, acting on diphenols and related substances as donors | molecular_function | 0.001034 |
| GO:0018995 | host | cellular_component | 0.001034 |
| GO:0043245 | extraorganismal space | cellular_component | 0.001034 |
| GO:0043657 | host cell | cellular_component | 0.001034 |
| GO:0044215 | other organism | cellular_component | 0.001034 |
| GO:0044216 | other organism cell | cellular_component | 0.001034 |
| GO:0044217 | other organism part | cellular_component | 0.001034 |
| GO:0006139 | nucleobase-containing compound metabolic process | biological_process | 0.0010931 |
| GO:0008150 | biological_process | biological_process | 0.0012773 |
| GO:0008104 | protein localization | biological_process | 0.0013945 |
| GO:0016491 | oxidoreductase activity | molecular_function | 0.0014213 |
| GO:0000228 | nuclear chromosome | cellular_component | 0.0014521 |
| GO:0051641 | cellular localization | biological_process | 0.0015643 |
| GO:0044454 | nuclear chromosome part | cellular_component | 0.0019978 |
| GO:0043168 | anion binding | molecular_function | 0.0019978 |
| GO:0044422 | organelle part | cellular_component | 0.0020855 |
| GO:0044446 | intracellular organelle part | cellular_component | 0.0028125 |
| GO:0019222 | regulation of metabolic process | biological_process | 0.0028125 |
| GO:0036094 | small molecule binding | molecular_function | 0.0030126 |
| GO:0031323 | regulation of cellular metabolic process | biological_process | 0.0037248 |
| GO:0044421 | extracellular region part | cellular_component | 0.0037466 |
| GO:0048519 | negative regulation of biological process | biological_process | 0.0046207 |
| GO:0006091 | generation of precursor metabolites and energy | biological_process | 0.0051483 |
| GO:0090304 | nucleic acid metabolic process | biological_process | 0.0051483 |
| GO:0030554 | adenyl nucleotide binding | molecular_function | 0.0053794 |
| GO:0005976 | polysaccharide metabolic process | biological_process | 0.0053794 |
| GO:0000794 | condensed nuclear chromosome | cellular_component | 0.0056855 |
| GO:0080090 | regulation of primary metabolic process | biological_process | 0.0057256 |
| GO:0032559 | adenyl ribonucleotide binding | molecular_function | 0.0058099 |
| GO:0000166 | nucleotide binding | molecular_function | 0.0060706 |
| GO:1901265 | nucleoside phosphate binding | molecular_function | 0.0060706 |
| GO:0051649 | establishment of localization in cell | biological_process | 0.0061176 |
| GO:0015031 | protein transport | biological_process | 0.0063765 |
| GO:0005524 | ATP binding | molecular_function | 0.0065607 |
| GO:1901564 | organonitrogen compound metabolic process | biological_process | 0.0065607 |
| GO:1901566 | organonitrogen compound biosynthetic process | biological_process | 0.0067157 |
| GO:0045184 | establishment of protein localization | biological_process | 0.0067571 |
| GO:0005634 | nucleus | cellular_component | 0.0067708 |
| GO:0000271 | polysaccharide biosynthetic process | biological_process | 0.0067708 |
| GO:0044264 | cellular polysaccharide metabolic process | biological_process | 0.0067708 |
| GO:0042025 | host cell nucleus | cellular_component | 0.0068887 |
| GO:0006508 | proteolysis | biological_process | 0.0075559 |
| GO:0008238 | exopeptidase activity | molecular_function | 0.0088688 |
| GO:0005044 | scavenger receptor activity | molecular_function | 0.0091105 |
| GO:0038024 | cargo receptor activity | molecular_function | 0.0091105 |
| GO:0006265 | DNA topological change | biological_process | 0.0096504 |
| GO:0000793 | condensed chromosome | cellular_component | 0.0098122 |
| GO:0051704 | multi-organism process | biological_process | 0.011309 |
| GO:0032991 | macromolecular complex | cellular_component | 0.011439 |
| GO:0043248 | proteasome assembly | biological_process | 0.012044 |
| GO:0051701 | interaction with host | biological_process | 0.012853 |
| GO:0004252 | serine-type endopeptidase activity | molecular_function | 0.012889 |
| GO:0033647 | host intracellular organelle | cellular_component | 0.013065 |
| GO:0033648 | host intracellular membrane-bounded organelle | cellular_component | 0.013065 |
| GO:0019219 | regulation of nucleobase-containing compound metabolic process | biological_process | 0.013529 |
| GO:0051171 | regulation of nitrogen compound metabolic process | biological_process | 0.013529 |
| GO:0033646 | host intracellular part | cellular_component | 0.013726 |
| GO:0043656 | intracellular region of host | cellular_component | 0.013726 |
| GO:0033692 | cellular polysaccharide biosynthetic process | biological_process | 0.014096 |
| GO:0060255 | regulation of macromolecule metabolic process | biological_process | 0.015517 |
| GO:0006790 | sulfur compound metabolic process | biological_process | 0.015827 |
| GO:0016829 | lyase activity | molecular_function | 0.016102 |
| GO:0048523 | negative regulation of cellular process | biological_process | 0.016552 |
| GO:0051726 | regulation of cell cycle | biological_process | 0.016553 |
| GO:0022900 | electron transport chain | biological_process | 0.016687 |
| GO:0009119 | ribonucleoside metabolic process | biological_process | 0.018879 |
| GO:0010468 | regulation of gene expression | biological_process | 0.021897 |
| GO:0017076 | purine nucleotide binding | molecular_function | 0.023415 |
| GO:0009110 | vitamin biosynthetic process | biological_process | 0.023547 |
| GO:0042364 | water-soluble vitamin biosynthetic process | biological_process | 0.023547 |
| GO:0005856 | cytoskeleton | cellular_component | 0.025253 |
| GO:0032555 | purine ribonucleotide binding | molecular_function | 0.026278 |
| GO:0044723 | single-organism carbohydrate metabolic process | biological_process | 0.026278 |
| GO:0032553 | ribonucleotide binding | molecular_function | 0.026278 |
| GO:0044281 | small molecule metabolic process | biological_process | 0.027666 |
| GO:0001883 | purine nucleoside binding | molecular_function | 0.027666 |
| GO:0032549 | ribonucleoside binding | molecular_function | 0.027666 |
| GO:0032550 | purine ribonucleoside binding | molecular_function | 0.027666 |
| GO:0034613 | cellular protein localization | biological_process | 0.027666 |
| GO:0070727 | cellular macromolecule localization | biological_process | 0.027666 |
| GO:0006766 | vitamin metabolic process | biological_process | 0.027666 |
| GO:0006767 | water-soluble vitamin metabolic process | biological_process | 0.027666 |
| GO:0044262 | cellular carbohydrate metabolic process | biological_process | 0.028092 |
| GO:0043169 | cation binding | molecular_function | 0.028258 |
| GO:0035639 | purine ribonucleoside triphosphate binding | molecular_function | 0.031191 |
| GO:0044271 | cellular nitrogen compound biosynthetic process | biological_process | 0.031541 |
| GO:0008608 | attachment of spindle microtubules to kinetochore | biological_process | 0.031541 |
| GO:0001882 | nucleoside binding | molecular_function | 0.031541 |
| GO:0016740 | transferase activity | molecular_function | 0.032773 |
| GO:0009058 | biosynthetic process | biological_process | 0.035859 |
| GO:0009987 | cellular process | biological_process | 0.035859 |
| GO:0046907 | intracellular transport | biological_process | 0.036226 |
| GO:0016820 | hydrolase activity, acting on acid anhydrides, catalyzing transmembrane movement of substances | molecular_function | 0.036791 |
| GO:0019538 | protein metabolic process | biological_process | 0.03706 |
| GO:0051252 | regulation of RNA metabolic process | biological_process | 0.03706 |
| GO:0046872 | metal ion binding | molecular_function | 0.03706 |
| GO:0000781 | chromosome, telomeric region | cellular_component | 0.03706 |
| GO:0016798 | hydrolase activity, acting on glycosyl bonds | molecular_function | 0.038909 |
| GO:1901362 | organic cyclic compound biosynthetic process | biological_process | 0.038909 |
| GO:0030246 | carbohydrate binding | molecular_function | 0.039881 |
| GO:0004553 | hydrolase activity, hydrolyzing O-glycosyl compounds | molecular_function | 0.040977 |
| GO:0010556 | regulation of macromolecule biosynthetic process | biological_process | 0.040977 |
| GO:0031326 | regulation of cellular biosynthetic process | biological_process | 0.040977 |
| GO:0009889 | regulation of biosynthetic process | biological_process | 0.041829 |
| GO:0034637 | cellular carbohydrate biosynthetic process | biological_process | 0.042726 |
| GO:0018130 | heterocycle biosynthetic process | biological_process | 0.043825 |
| GO:2000112 | regulation of cellular macromolecule biosynthetic process | biological_process | 0.045029 |
| GO:0006073 | cellular glucan metabolic process | biological_process | 0.045029 |
| GO:0044042 | glucan metabolic process | biological_process | 0.045029 |
| GO:0005737 | cytoplasm | cellular_component | 0.045029 |
| GO:0034453 | microtubule anchoring | biological_process | 0.045245 |
| GO:0000778 | condensed nuclear chromosome kinetochore | cellular_component | 0.047 |
| GO:0000780 | condensed nuclear chromosome, centromeric region | cellular_component | 0.047 |
| GO:0000942 | condensed nuclear chromosome outer kinetochore | cellular_component | 0.047 |
| GO:0042729 | DASH complex | cellular_component | 0.047 |

**Supplemental Table 7.** The enriched Kyoto Encyclopedia of Genes and Genomes pathways for mRNA targets of differentially expressed miRNA from PN1 (gestational d 85) v PN2 (gestational d 110) comparison.

| **ID** | **Pathway** | **Corrected P-Value** |
| --- | --- | --- |
| oas05140 | Leishmaniasis | 0.01393294 |
| oas04145 | Phagosome | 0.02208794 |
| oas05150 | Staphylococcus aureus infection | 0.02208794 |
| oas04210 | Apoptosis | 0.02208794 |
| oas04141 | Protein processing in endoplasmic reticulum | 0.02208794 |
| oas04142 | Lysosome | 0.02208794 |
| oas04612 | Antigen processing and presentation | 0.02208794 |
| oas05145 | Toxoplasmosis | 0.02208794 |
| oas01100 | Metabolic pathways | 0.02208794 |
| oas03050 | Proteasome | 0.02762943 |
| oas05330 | Allograft rejection | 0.03364853 |
| oas04152 | AMPK signaling pathway | 0.03446092 |
| oas05168 | Herpes simplex infection | 0.0347534 |
| oas05202 | Transcriptional misregulation in cancer | 0.0347534 |
| oas05200 | Pathways in cancer | 0.03603378 |
| oas00562 | Inositol phosphate metabolism | 0.04172398 |

**Supplemental Table 8.** The enriched Kyoto Encyclopedia of Genes and Genomes pathways for mRNA targets of differentially expressed miRNA from PN2 (gestational d 110) v PN3 (gestational d 133) comparison.

| **ID** | **Pathway** | **Corrected P-Value** |
| --- | --- | --- |
| oas04142 | Lysosome | 0.00109482 |
| oas04141 | Protein processing in endoplasmic reticulum | 0.00204419 |
| oas05140 | Leishmaniasis | 0.00204419 |
| oas01100 | Metabolic pathways | 0.00204419 |
| oas04145 | Phagosome | 0.00256629 |
| oas04976 | Bile secretion | 0.01336534 |
| oas05168 | Herpes simplex infection | 0.01336534 |
| oas05152 | Tuberculosis | 0.01336534 |
| oas05200 | Pathways in cancer | 0.01336534 |
| oas05150 | Staphylococcus aureus infection | 0.01802361 |
| oas03050 | Proteasome | 0.01933322 |
| oas04210 | Apoptosis | 0.02479498 |
| oas05146 | Amoebiasis | 0.02688592 |
| oas05145 | Toxoplasmosis | 0.02688592 |
| oas05202 | Transcriptional misregulation in cancer | 0.03209157 |
| oas04151 | PI3K-Akt signaling pathway | 0.04482981 |
| oas04612 | Antigen processing and presentation | 0.0470291 |

**Supplemental Table 9.** The enriched Kyoto Encyclopedia of Genes and Genomes pathways for mRNA targets of differentially expressed miRNA from PN3 (gestational d 133) v PW1 (d 42) comparison.

| **ID** | **Pathway** | **Corrected P-Value** |
| --- | --- | --- |
| oas04145 | Phagosome | 0.00157811 |
| oas04141 | Protein processing in endoplasmic reticulum | 0.00309986 |
| oas05140 | Leishmaniasis | 0.00309986 |
| oas05150 | Staphylococcus aureus infection | 0.00309986 |
| oas04976 | Bile secretion | 0.00309986 |
| oas04142 | Lysosome | 0.00309986 |
| oas05200 | Pathways in cancer | 0.00309986 |
| oas05152 | Tuberculosis | 0.00380025 |
| oas01100 | Metabolic pathways | 0.0045276 |
| oas05168 | Herpes simplex infection | 0.0045276 |
| oas04612 | Antigen processing and presentation | 0.0105796 |
| oas04970 | Salivary secretion | 0.0105796 |
| oas05202 | Transcriptional misregulation in cancer | 0.02882063 |
| oas03050 | Proteasome | 0.03268771 |
| oas04210 | Apoptosis | 0.03268771 |
| oas05146 | Amoebiasis | 0.03389536 |
| oas04916 | Melanogenesis | 0.03389536 |
| oas04540 | Gap junction | 0.03389536 |
| oas05134 | Legionellosis | 0.03453992 |
| oas02010 | ABC transporters | 0.0486229 |
| oas04810 | Regulation of actin cytoskeleton | 0.0486229 |
| oas04610 | Complement and coagulation cascades | 0.05055773 |
| oas04151 | PI3K-Akt signaling pathway | 0.05055773 |
| oas04014 | Ras signaling pathway | 0.05435586 |

**Supplemental Table 10.** The enriched Kyoto Encyclopedia of Genes and Genomes pathways for mRNA targets of differentially expressed miRNA from PW1 (d 42) v PW2 (d 65) comparison.

| **ID** | **Pathway** | **Corrected P-Value** |
| --- | --- | --- |
| oas04974 | Protein digestion and absorption | 0.00415388 |
| oas03050 | Proteasome | 0.02310997 |

**Supplemental Table 11.** The enriched Kyoto Encyclopedia of Genes and Genomes pathways for mRNA targets of differentially expressed miRNA from PW2 (d 65) v MAT (d 203) comparison.

| **ID** | **Pathway** | **Corrected P-Value** |
| --- | --- | --- |
| oas05140 | Leishmaniasis | 0.0013265 |
| oas05150 | Staphylococcus aureus infection | 0.00224321 |
| oas04152 | AMPK signaling pathway | 0.00224321 |
| oas05330 | Allograft rejection | 0.00863106 |
| oas04141 | Protein processing in endoplasmic reticulum | 0.0117168 |
| oas05332 | Graft-versus-host disease | 0.01686354 |
| oas04145 | Phagosome | 0.01686354 |
| oas04210 | Apoptosis | 0.01686354 |
| oas05145 | Toxoplasmosis | 0.01686354 |
| oas04612 | Antigen processing and presentation | 0.01686354 |
| oas05321 | Inflammatory bowel disease (IBD) | 0.01686354 |
| oas03050 | Proteasome | 0.02945817 |
| oas05168 | Herpes simplex infection | 0.02945817 |
| oas04940 | Type I diabetes mellitus | 0.02945817 |
| oas04672 | Intestinal immune network for IgA production | 0.02987292 |
| oas05310 | Asthma | 0.03482687 |
| oas05320 | Autoimmune thyroid disease | 0.03482687 |
| oas05164 | Influenza A | 0.03831313 |
| oas05152 | Tuberculosis | 0.04551235 |
| oas04150 | mTOR signaling pathway | 0.04551235 |
| oas05202 | Transcriptional misregulation in cancer | 0.04551235 |
